# De novo Biosynthesis of “Non‐Natural” Thaxtomin Phytotoxins

**DOI:** 10.1002/anie.201801525

**Published:** 2018-05-08

**Authors:** Michael Winn, Daniel Francis, Jason Micklefield

**Affiliations:** ^1^ School of Chemistry Manchester Institute of Biotechnology The University of Manchester 131 Princess Street Manchester M1 7DN UK

**Keywords:** biocatalysis, biosynthesis, biosynthetic engineering, nonribosomal peptides, synthetic biology

## Abstract

Thaxtomins are diketopiperazine phytotoxins produced by *Streptomyces scabies* and other actinobacterial plant pathogens that inhibit cellulose biosynthesis in plants. Due to their potent bioactivity and novel mode of action there has been considerable interest in developing thaxtomins as herbicides for crop protection. To address the need for more stable derivatives, we have developed a new approach for structural diversification of thaxtomins. Genes encoding the thaxtomin NRPS from *S. scabies*, along with genes encoding a promiscuous tryptophan synthase (TrpS) from *Salmonella typhimurium*, were assembled in a heterologous host *Streptomyces albus*. Upon feeding indole derivatives to the engineered *S. albus* strain, tryptophan intermediates with alternative substituents are biosynthesized and incorporated by the NRPS to deliver a series of thaxtomins with different functionalities in place of the nitro group. The approach described herein, demonstrates how genes from different pathways and different bacterial origins can be combined in a heterologous host to create a de novo biosynthetic pathway to “non‐natural” product target compounds.

Many valuable therapeutic agents and agrochemicals are derived from natural products. However, many of these compounds do not have the necessary bioactivity or physicochemical properties at the outset, and further derivatization or analogue synthesis is required to develop optimized, effective compounds. This optimization is traditionally achieved through semi‐synthesis or total synthesis. However, multistep chemical synthesis often requires deleterious reagents and can be costly, which is particularly problematic for the development of agrochemicals that need to be produced in large quantities. Biosynthetic engineering is an alternative approach to produce new and potentially improved natural product variants directly by fermentation.^1^, ^2^ Although biosynthetic engineering has been used to generate novel “non‐natural” products^1^, ^2^, ^3^, ^4^, ^5^, ^6^ most approaches have been limited to manipulating biosynthetic pathways in the well characterized native producers. However there are many microorganisms that produce useful secondary metabolites, which are not amenable to such genetic manipulation. The rise of synthetic biology has provided access to larger synthetic DNA constructs, rapid DNA capture,^7^, ^8^, ^9^ editing,^10^, ^11^ assembly,^12^, ^13^ and other advances.^14^, ^15^ The prospect of using these new tools to assemble de novo biosynthetic pathways in well‐characterized heterologous host strains^16^, ^17^ for diversification and optimization of natural products, derived from less tractable microorganisms, is an attractive goal.

In this paper we describe a new approach for the de novo biosynthesis of “non‐natural” variants of thaxtomin phytotoxins produced by *Streptomyces scabies* (Figure 1 A), which inhibit cellulose biosynthesis in plants.^18^, ^19^ Thaxtomins (**1**–**11**) are biosynthesized from l‐phenylalanine and the unusual l‐4‐nitrotryptophan, which is produced by TxtD and TxtE enzymes (Figure 1 B).^20^, ^21^, ^22^, ^23^ TxtD is a nitric oxide (NO) synthase and TxtE, a P450 enzyme, then utilizes this NO with O_2_, to catalyze nitration at the 4‐position of l‐tryptophan.^21^, ^24^ Nonribosomal peptide synthetases (NRPS) TxtA and TxtB activate the l‐phenylalanine and l‐4‐nitrotryptophan precursors, catalyzing subsequent condensation, N‐methylation and cyclisation to give diketopiperazines including thaxtomin D (**3**). A P450 monooxygenase, TxtC, then hydroxylates the l‐phenylalanine residue at the α‐position and at either 2‐, 3‐, or 4‐positions of the aryl group resulting in a family of compounds (**4**–**11**) with thaxtomin A (**6**) being the major metabolite produced by *S. scabies*.^25^, ^26^, ^27^, ^28^ The potent herbicidal activity and novel mode of action has driven the development of several synthetic routes to thaxtomins.^29^, ^30^, ^31^, ^32^, ^33^, ^34^, ^35^, ^36^ However, these routes are all multi‐step, low yielding, and produce diastereoisomeric mixtures. We therefore sought to establish an alternative biosynthetic route for the production of thaxtomin derivatives. Previous studies have established that the 4‐nitro substituent of the l‐tryptophan is important for bioactivity. However this moiety is photolabile and can result in harmful degradation products.^37^, ^38^ Consequently, thaxtomin derivatives with alternative substituents at the C4 position of the indole ring were identified as target compounds for synthetic biology.

**Figure 1:**
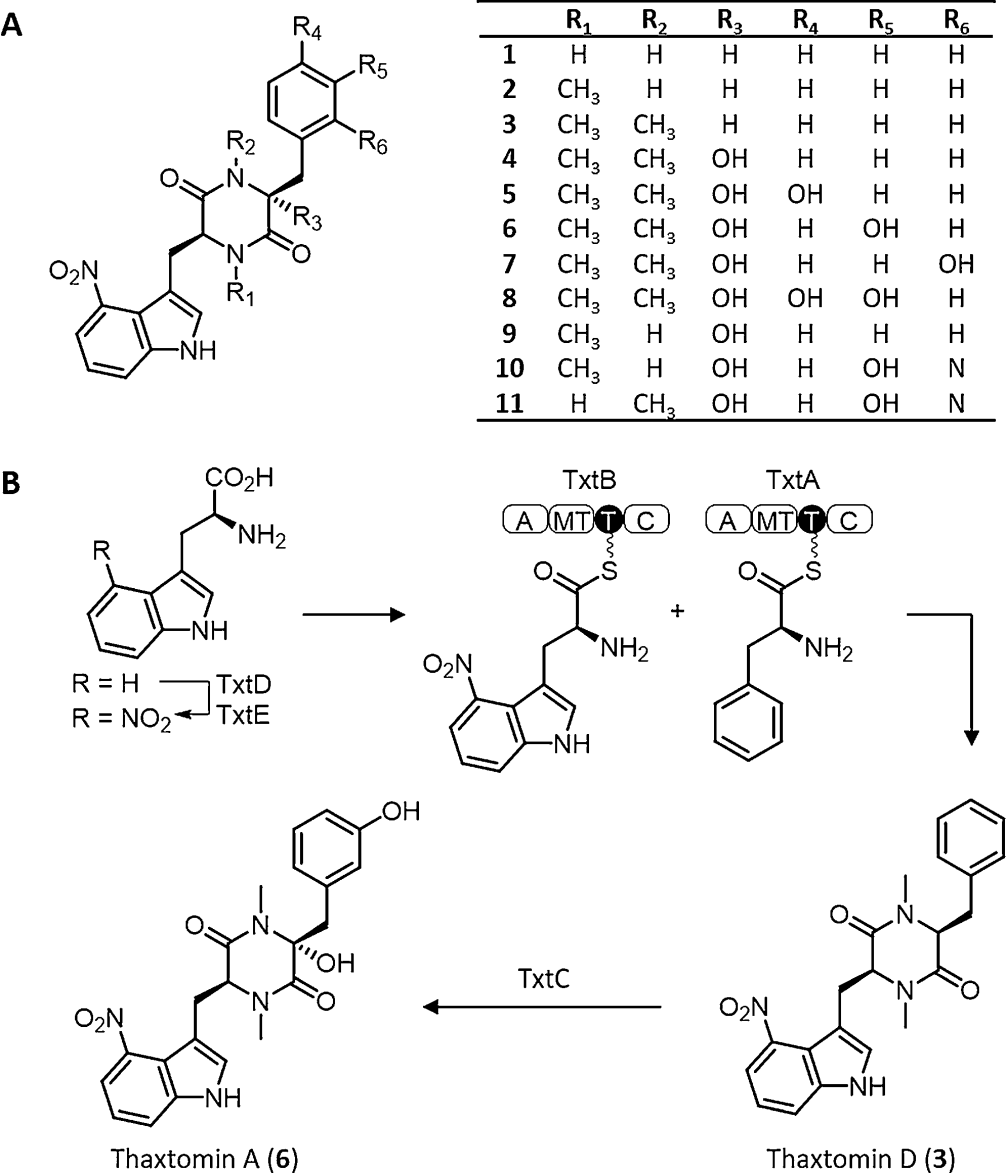
Thaxtomin biosynthesis. A) Thaxtomin congeners from *Streptomyces scabies*. B) The proposed biosynthesis of thaxtomins.

In addition to being difficult to genetically manipulate, *S. scabies* and other thaxtomin producers are plant pathogens with tight regulations controlling their use and genetic modification. It was thus desirable to establish heterologous expression of thaxtomin biosynthetic genes in a more tractable strain. To this end, *Streptomyces albus* was selected as a suitable host. Initially a two‐step process for production of thaxtomin derivatives was envisaged; a biotransformation step utilizing tryptophan synthase (TrpS) to produce 4‐substituted l‐tryptophans (Figure 2), which are then fed to an *S. albus* strain possessing the NRPS TxtA and TxtB (Figure 3). Previous studies have shown that the TrpS from *Salmonella typhimurium* (ATCC 37845) is promiscuous and will condense a range of indole derivatives with l‐serine or l‐threonine to yield l‐Trp derivatives.^39^, ^40^, ^41^, ^42^ To explore if this enzyme could be used to generate a library of C4‐substituted tryptophan analogues, *St*TrpS was overproduced in *E. coli* BL21 and the resulting cell lysate incubated with a variety of 4‐substituted indoles. The resulting tryptophan analogues were isolated with yields ranging from 5–49 % (Figure 2). For the second step in the process (Figure 3), NRPS‐encoding genes *txtA* and *txtB* were amplified from *S. scabies* genomic DNA as two separate DNA parts, with optimized ribosomal binding sites and spacer sequences replacing the native RBS and spacer sequences of each gene. These two DNA parts were then assembled into a non‐integrative vector under the control of the constitutive ermE* promoter. This vector was conjugated into *S. albus* and the resulting strain (Figure 3, i) was supplemented with l‐4‐nitrotryptophan (**12**). After 3 days of fermentation the cells were removed and the culture supernatant was purified by C18 RP‐HPLC affording thaxtomin D (**3**).

**Figure 2:**
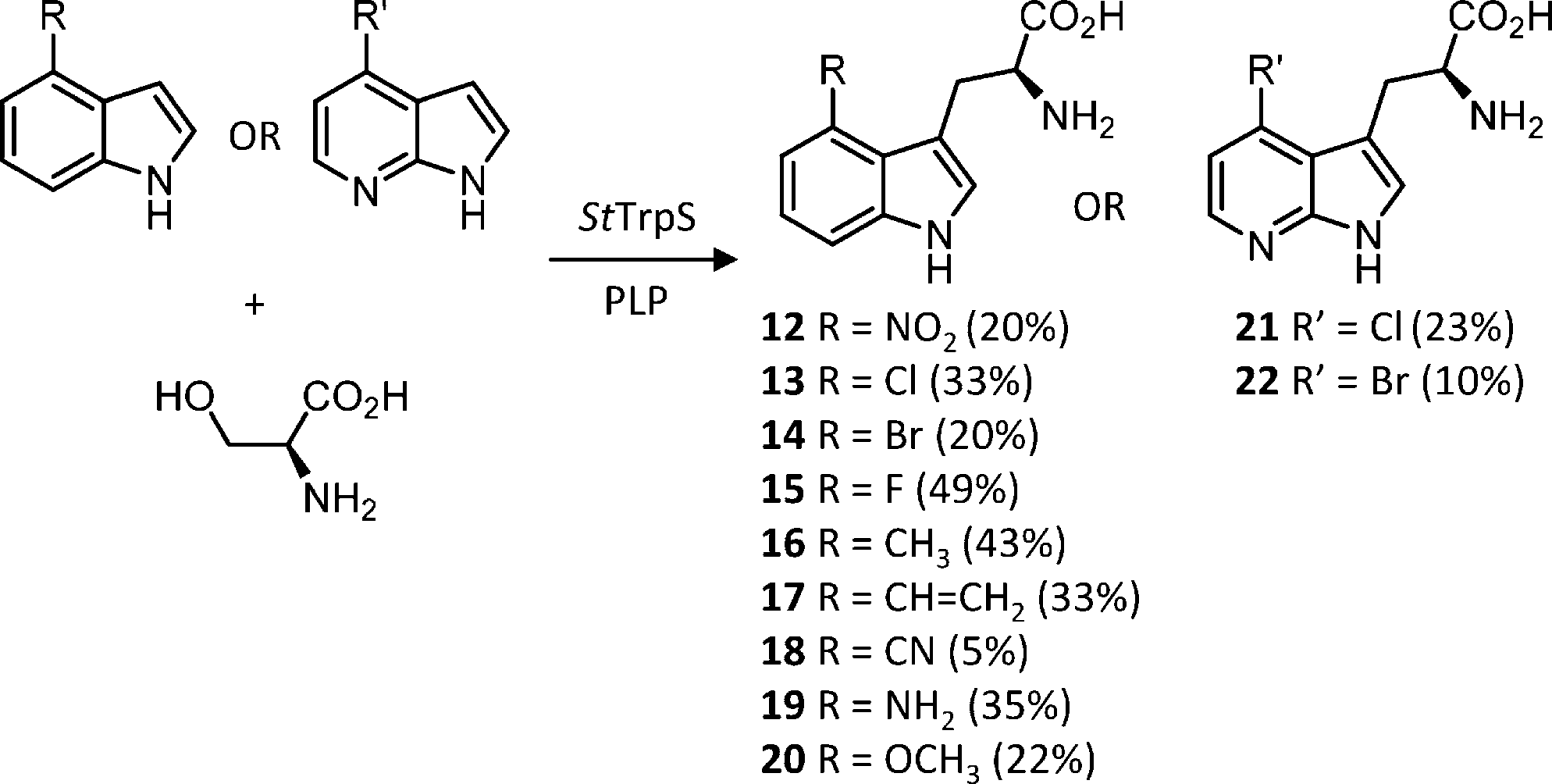
Enzymatic synthesis of tryptophan derivatives using tryptophan synthase from *Salmonella typhimurium* (*St*TrpS). Reactions were carried out in 50 mL with 15 mm indole analogue, 30 mm l‐Ser, 0.25 mm PLP, 100 mm KPi pH 7.6, 5 mL *St*TrpS cell lysate and incubated at 37 °C for 72 h.

**Figure 3:**
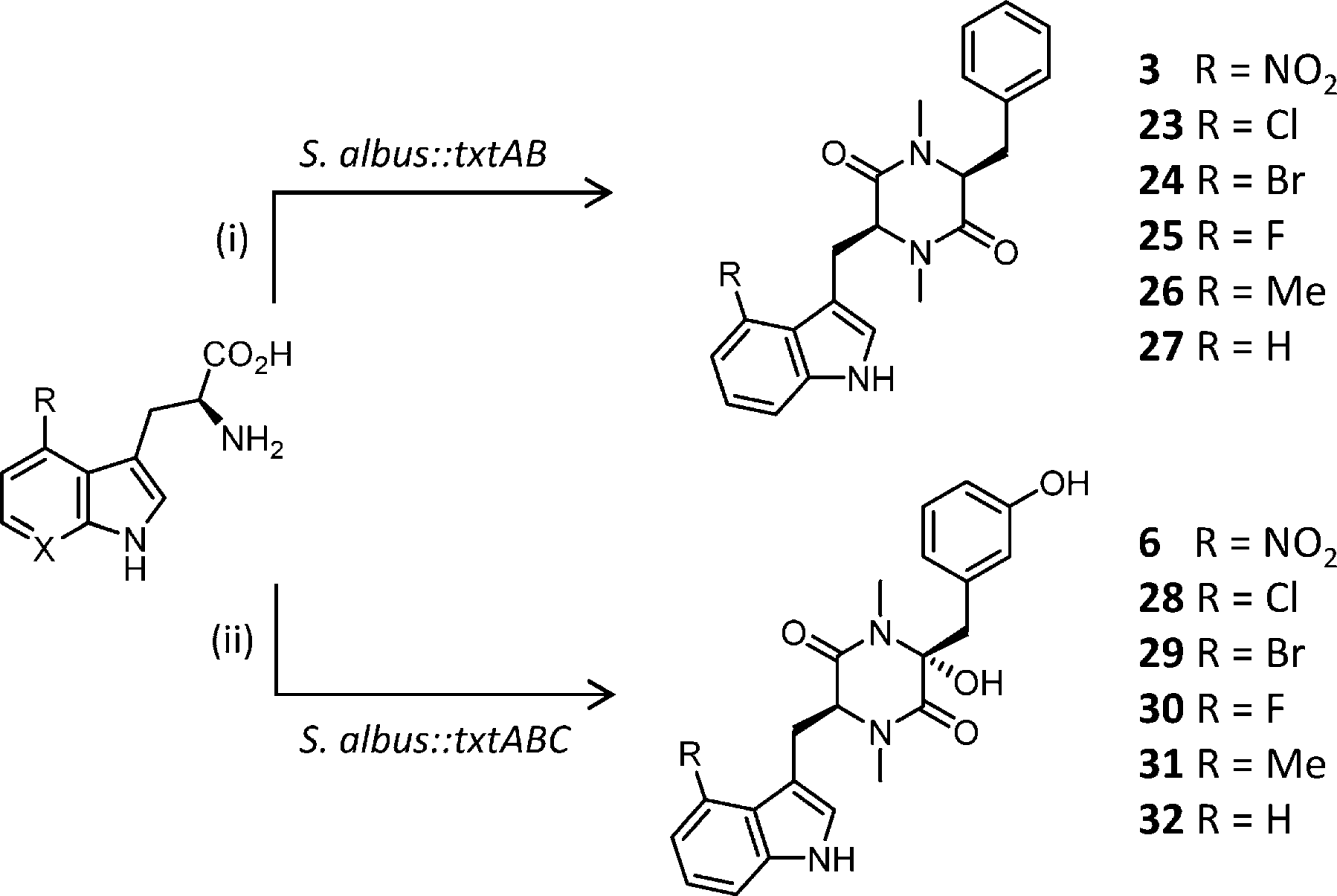
Heterologous biosynthesis of thaxtomin derivatives from modified tryptophans. i) The two NRPS genes required for thaxtomin biosynthesis in *S. scabies* were assembled into a synthetic operon and heterologously expressed in *S. albus*. Feeding of tryptophan analogues to this strain resulted in new thaxtomin D analogues. ii) The inclusion of the P450 TxtC into the pathway resulted in hydroxylated thaxtomin A analogues.

The *S. albus::txtAB* strain was then supplemented with the tryptophan derivatives **13**–**20** which resulted in new thaxtomin D analogues possessing Cl, Br, F, or Me substituents at C4 of the indole moiety (**23**–**26**) (Figure 3, Table S1 and Figure S1A in the Supporting Information). Interestingly, other than 4‐nitrotryptophan, none of the tryptophan analogues possessing more polar substituents were incorporated. This could be due to substrate selectivity of the thaxtomin NRPS and/or less efficient cellular uptake of the more polar l‐Trp derivatives. To explore if the structural diversity of thaxtomin derivatives could be further expanded, 4‐chloro‐ and 4‐bromo‐7‐azatryptophans (**21** & **22**) were prepared (Figure 2) and fed to *S. albus:txtAB*. Whilst, 4‐chloro‐ and 4‐bromotryptophan (**13** & **14**) are accepted, the corresponding azatryptophans (**21** & **22**), which are the same size but more polar, are not incorporated which further suggests that hydrophobicity rather than steric factors appear to govern selectivity of the NRPS. In addition to the five thaxtomin D analogues, a new thaxtomin containing unmodified tryptophan (**27**) was also detected in each of the cultures, including those that were not supplemented with exogenous precursors. There is no evidence in the literature of desnitro thaxtomins being produced by *S. scabies*, and none were detected in cultures of *S. scabies* grown for this study. Presumably in the wild type *S. scabies* l‐4‐nitrotryptophan is present in abundance during thaxtomin production, which prevents TxtB loading l‐tryptophan. Another strain, *Streptomyces ipomoeae*, is reported to make desnitro thaxtomin(s), this strain may be less efficient in producing l‐4‐nitrotryptophan and/or may possess a more promiscuous NRPS assembly line.^43^

While thaxtomin D (**3**) is known to possess some herbicidal activity, the hydroxylated thaxtomin A (**6**) is a more potent herbicide, mostly due to a gain in solubility rather than any direct increase in biological activity.^28^ To this end a third DNA part, with optimized ribosomal binding and spacer sequence, was constructed by amplifying the thaxtomin hydroxylase *txtC* from *S. scabies* genomic DNA. All three DNA parts (*txtA*, *txtB* and *txtC*) were then assembled together into a synthetic operon, within the non‐integrative vector, under the control of the ermE* promoter and introduced into the *S. albus* host. The resulting strain *S. albus::txtABC* (Figure 3, ii) was cultivated and supplemented with 4‐nitrotryptophan, which resulted in a major product with *m*/*z* 439.1616, which has an identical HPLC retention time and MS‐MS to a sample of thaxtomin A (**6**) (Δ 0.4 ppm), which was isolated from *S. scabies* (Table S2, Figure S2). Two other minor products (<10 %) were also evident with *m*/*z* 439.1623 and 439.1621 (Δ 1 and 0.8 ppm), which are most likely regioisomers that have undergone hydroxylation at the α‐position and at C2 or C4 of the l‐phenylalanine aryl group. These additional two peaks are also present in samples of thaxtomin A isolated from *S. scabies* with identical retention times and MS fragmentation patterns (Figure S2). It has previously been shown that whilst thaxtomin A is the main metabolite produced by *S. scabies* minor congeners that have undergone hydroxylation at C2 or C4 (**5** & **7**, Figure 1 A) can also be isolated.^44^ Taken together with the results presented here, this suggest that the P450 hydroxylase TxtC has relaxed regioselectivity. In addition, *S. albus:txtABC* supplemented with 4‐nitrotryptophan also produced minor amounts of a metabolite with *m*/*z* 407.1710, which was identical to thaxtomin D (**3**)(Δ 0.3 ppm), and a metabolite *m*/*z* 423.1667, consistent with the mono‐hydroxylated derivative **4** (Δ 0.4 ppm) (Figure S3). It has previously been postulated that TxtC first hydroxylates the α‐carbon of the phenylalanine, before introducing a second hydroxyl group on the aromatic ring as only thaxtomins singly hydroxylated at the α position have been isolated from the wild type strain (compound **4** and **9**, Figure 1 A).^44^

Having established that *S. albus::txtABC* (Figure 3, ii) can produce hydroxylated thaxtomin A, this strain was then supplemented with tryptophan derivatives **13**–**16**. This resulted in new hydroxylated thaxtomin analogues with either Cl, Br, F, or Me group at the 4‐position of the indole ring. As with the natural pathway, the cultures fed with the three halogenated tryptophan analogues (**13**–**15**) also resulted in several dihydroxylated thaxtomins variants, with a single regioisomer forming preferentially (>75 %, Figure S4 i–iv, Table S2). Some residual non hydroxylated and singly hydroxylated intermediates could also be detected in each case. Methylated and desnitro thaxtomins (compounds **26** and **27**) were also converted by TxtC into hydroxylated analogues, however these two substrates resulted in the formation of a singly hydroxylated compound predominantly with around 25 % conversion to the major doubly hydroxylated product (Figure S4 v,vi).

It was then envisaged that a third strain could be developed that was capable of producing thaxtomin analogues directly from simple, commercially available indole starting materials by introducing the tryptophan synthase genes, *trpA* and *trpB*, from *S. typhimurium*, along with genes encoding the thaxtomin NRPS, *txtA* and *txtB*, into the *S. albus* host (Figure 4). Using 4‐substituted indole as the starting material would also provide more lipophilic substrates that may promote better cellular uptake than the more polar tryptophan precursors. The *trpA* and *trpB* genes from *S. typhimurium* were optimized for codon usage in *Streptomyces* and used to create two further DNA parts with ribosome binding and spacer sequences optimized which were then assembled together with the *S. scabies txtA* and *txtB* into the non‐integrative ermE*p containing vector used before and conjugated into the *S. albus* host. Cultures of this exconjugant (*S. albus::txtAB,trpAB*, Figure 4) were supplemented with commercially available indole analogues. Following several days incubation, thaxtomin D (**3**) and analogues possessing Br, Cl, F, and Me substituents (**23**–**26**) were again produced. In contrast, control experiments, where indole analogues are fed to *S. albus::txtAB* or *S. albus::txtABC* strains lacking the heterologous *trpAB,* produced only trace amounts of thaxtomin analogues (Figure S6 and S7), which illustrates the success of this de novo pathway (Figure S5). The fact that *S. albus::txtAB,trpAB* failed to produce thaxtomin derivatives from the indoles with polar substituents, despite the increased lipophilicity of the indole versus tryptophan, indicates that cellular uptake may not be the reason why these are not accepted as substrates by the pathway, instead A‐domain specificity may be limiting analogue incorporation.

**Figure 4:**
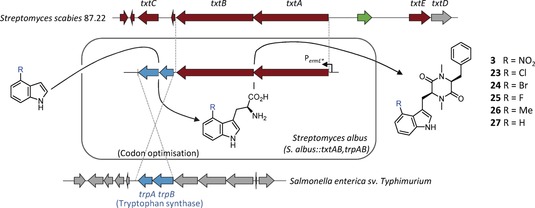
A synthetic pathway for biosynthesis of thaxtomin D derivatives directly from indole. The two NRPS genes required for thaxtomin biosynthesis in *S. scabies* were optimized into a synthetic operon with codon optimized TrpS genes from *Salmonella* and heterologously expressed in *S. albus*. Feeding of indole analogues to this strain resulted in thaxtomin D analogues being produced in vivo.

In summary, we have assembled a novel artificial pathway that allows thaxtomin analogues to be generated in vivo in a single fermentation step from indole precursors. TrpS has been widely used to generate tryptophan analogues in vitro, however the work described here shows how TrpS can also be exploited to generate tryptophan derivatives in vivo providing alternative precursors for incorporation into natural products. Given that there are many natural products derived from tryptophan, this approach could be adopted to create a wide range of new biosynthetic assembly lines, in vivo, generating novel natural product variants. Consequently, in addition to providing new ways of producing more effective herbicides, this approach could also be applied to diversify a wide range of other bioactive natural product scaffolds including important antibiotics. One can envisage expanding this concept, of combining multiple enzymes from different pathways and origins in heterologous host strains, to create many novel “non‐natural” variants for agrochemical, therapeutic and other applications.

## Conflict of interest

The authors declare no conflict of interest.

## Supporting information

As a service to our authors and readers, this journal provides supporting information supplied by the authors. Such materials are peer reviewed and may be re‐organized for online delivery, but are not copy‐edited or typeset. Technical support issues arising from supporting information (other than missing files) should be addressed to the authors.

Supplementary
