# Supplementary material for: De novo Biosynthesis of “Non‐Natural” Thaxtomin Phytotoxins
Source: Angew Chem Int Ed Engl. 2018 May 8;57(23):6830–3. doi: 10.1002/anie.201801525 (PMC6001691; doi:10.1002/anie.201801525)
Supplement: Supplementary file 1 — Supplementary [file ANIE-57-6830-s001.pdf]

Angewandte Chemie
Eine Zeitschrift der Gesellschaft Deutscher Chemiker

Supporting Information

De novo Biosynthesis of "Non-Natural" Thaxtomin Phytotoxins

*Michael Winn, Daniel Francis, and Jason Micklefield**

anie_201801525_sm_miscellaneous_information.pdf

Supporting Information

Content:

1. Methods and Data from product characterisation (page S2-S7)
2. Supplementary Tables and Figures (page S8-S16)
3. NMR Spectra (page S17-S35)
4. Supplementary References and abbreviated references from manuscript (page S36)

Further Experimental Methods

Unless otherwise stated, all chemicals were purchased from Sigma-Aldrich, Fisher Scientific or Acros organics and used without further purification. NMR analysis was performed on a Bruker 400 MHz or 800 MHz spectrometer. Mass spectrometry was performed on an Agilent 6510 QTof mass spectrometer or a Waters Vion IMS QTof (tryptophan and thaxtomin analogues), or a Thermo Scientific Q-Exactive (HRMS LCMS) at the Michael Barber Centre for Mass Spectrometry and at the SynBioChem Centre (grant BB/M017702/1) at the University of Manchester.

Production of thaxtomin A from *S. scabies*. 10 mL of tryptone soya broth (TSB, Oxoid) was inoculated with 20 µL of a *S. scabies* 87.22 spore stock and grown for 48 hours at 28 °C in a 25 mL conical flask fitted with a spring. 1.5 mL of this seed culture was then used to inoculate 150 mL of thaxtomin production medium (TDMC)[1]. 1 litre of thaxtomin production medium consisted of 600 mL of autoclaved TDM1 (2 g $(NH_4)_2SO_4$, 0.6 g $MgSO_4.7H_2O$) added to 300 mL freshly autoclaved TDM2 (2.6 g K_2HPO_4), 100 mL filter sterilised cellubiose solution (10 g), and 1 mL of trace elements solution (1 g L^{-1} $FeSO_4.7H_2O$, $ZnSO_4.7H_2O$, $MnCl_2.4H_2O$, $CaCl_2$). The culture was incubated for 7 days, or until a strong yellow colour, representative of thaxtomin production, was seen. Centrifugation (4°C, 30 min, 10,000 g) removed the cells and the supernatant was loaded onto a pre-equilibrated C18 SPE cartridge (Mega BE-C18, 10 gm 60 mL, Agilent Technologies) and washed with 3 x 50 mL of H_2O. The thaxtomin was then eluted with 10 mL of MeOH and purified by RP-HPLC on a Luna AXIA C18 column (10 µM, 100Å, 250 x 21.2 mm) at 15 mL min^{-1} with a gradient of H_2O + 0.05% TFA versus (B) ACN 0.05% TFA (0-21 min 5-95% B, 21-25 min 95% B, 25-29 min 95-5% B, 29-33 min 5% B) to yield a yellow crystalline solid. **Thaxtomin A** (**6**): 1H NMR (400 MHz, MeOD) 7.74 (dd, J=7.8, 1.0 Hz,1H), 7.60 (dd, J= 8.1, 1.0 Hz,1H), 7.13 (t, J = 8 Hz, 1H), 7.09 (t, J = 8.0 Hz, 1H), 6.85 (s, 1H), 6.65 (ddd, J = 8.1, 2.4, 1.1 Hz, 1H), 6.60 (dt, J = 3.9, 1.7 Hz, 2H), 3.76 (dd, J = 8.8, 6.3 Hz, 1H), 3.02 (d, J = 13.5 Hz, 1H), 2.93 (s, 3H), 2.72 (s, 3H), 2.51 (dd, J = 14, 6.3 Hz, 1H), 1.51 (dd, J = 14.1, 8.8 Hz, 1H). HRMS (+ESI) *m/z*: calculated for $C_{22}H_{23}N_4O_6$ (M+H): 439.16121. Found 439.16169 (Δ 0.5 ppm).

Synthesis of 4-vinyl indole: A three necked round bottom flask was charged with 4-bromoindole (300 mg, 1.53 mmol), vinyl boronic acid pinicol ester (612 mg, 3.98 mmol) potassium carbonate (1.903 mg, 13.77 mmol) and tetrakis(triphenylphosphine)palladium(0) (176 mg, 0.153 mmol) in degassed water, ethanol and toluene (2:3:1 ratio, 30 mL). The solution was rapidly stirred at 80 ^{o}C overnight under nitrogen. The mixture was allowed to cool to room temperature and diluted with saturated ammonium chloride (30mL) and extracted with dichloromethane (3 x 60mL). Combined organics were dried over magnesium sulphate and solvent removed *in vacuo*. The crude material was purified using flash chromatography (0-10% EtOAc:Hexane) resulting in a viscous brown oil (75mg, 34%). ^{1}H NMR (400 MHz, CDCl3) δ 8.21 – 7.87 (s 1H NH), 7.19 (m,2H H7 H4), 7.10 (dt, J = 7.3, 2.6 Hz, 2H H2 H6), 7.08 – 7.01 (m, 1H C=CH-),), 6.67 (ddd, J = 3.0, 2.0, 0.9 Hz, 1H H3), 5.83 (dd, J = 17.7, 1.2 Hz, 1H =CH2), 5.30 (dd, J = 11.1, 1.2 Hz, 1H =CH2). ^{13}C NMR (101 MHz, CDCl3) δ 136.60 (C8), 135.81 (s), 130.46 (s), 126.43 (s), 124.87 (s), 122.49 (s), 117.81 (s), 115.08 (s), 111.02 (s), 101.54 (s), 77.79 (s), 77.48 (s), 77.34 – 77.03 (m). LRMS (+ESI) calculated for $C_{10}H_{10}N$ (M+H): 144.08. Found 144.1 (M+H).

Preparation of 4-tryptophan analogues. LB media (10 mL) (10 g L^{-1} tryptone, 10 g L^{-1} NaCl, 5 g L^{-1} yeast extract) supplemented with ampicillin (10 µg mL^{-1}) was inoculated with *E. coli* BL21 transformed with TrpS plasmid pSTB7 (ATCC 37845). Following 12 hours of growth (37°C) this seed culture was diluted into 400 mL of fresh LB and grown for a further 12 hours. The cells were collected by centrifugation (4°C, 15 min, 4000 g), washed with PBS and resuspended in 40 mL of buffer (0.1 M KH_2PO_4, 2.5 mM PLP, 7.5 mM EDTA, pH 7.4). Lysates were generated by sonication (10 mins, 50% cycle, 65% power) and cleared by centrifugation (4°C, 30 min, 10,000 g). Indole analogue (15 mM) was added to 50 mL potassium phosphate buffer (0.1 M KH_2PO_4, 2.5 mM PLP, 30 mM L-serine, pH 7.4), the reaction was then initiated by the addition of 5 mL of the pSTB7 cell lysate contained within dialysis membrane. Biotransformations were incubated for 72 hours (37°C). Following this the dialysis membrane was removed and unreacted indole extracted with EtOAc (3 x 50 mL). The aqueous layer was concentrated *in vacuo* and applied to a pre-equilibrated C18 SPE cartridge (Mega BE-C18, 10 gm 60 mL, Agilent Technologies) and washed with 3 x 50 mL of H_2O. The tryptophan analogue was then eluted with 10-20 mL of MeOH. Solvent was dried resulting in pure L-tryptophan analogues:

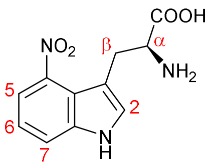

(12)

L-4-nitrotryptophan (12): ^{1}H NMR (400 MHz, D_2O) δ 7.87 (1H, d, *J* = 8.0 Hz, H5), 7.72 (1H, d, *J* = 8.1 Hz, H7), 7.41 (1H, s, H2), 7.16 (1H, t, *J* = 8.1 Hz, H6), 4.09 (1H, dd, *J* = 8.5, 6.0 Hz, Hα), 3.59 (1H, dd, *J* = 15.0, 5.8 Hz, Hβ’), 3.22 (1H, dd, *J* = 15.0, 8.7 Hz, Hβ’’). ^{13}C NMR (101 MHz, DMSO) δ 170.68 (C=O), 141.77 (C4), 139.55 (C8), 131.66 (C2), 120.13 (C6), 118.86 (C7), 118.43 (C5), 117.45 (C9), 106.54 (C3), 53.44 (Cα) (d, *J* = 21.0 Hz), 28.52 (Cβ).
HRMS (+ESI) *m/z*: calculated for $C_{11}H_{12}N_3O_4$ (M+H): 250.0822. Found 250.0829 (Δ 2.8 ppm).

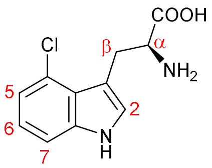

(**13**)

L-4-chlorotryptophan (13): ^{1}H NMR (400 MHz, D_2O) δ 7.40 (1H, dd, *J* = 6.9, 1.8 Hz, *H5*), 7.27 (1H, s, *H2*), 7.16 – 7.09 (2H, m, *H6 H7*), 4.38 (1H, dd, *J* = 9.5, 5.4 Hz, *Hα*), 3.81 (1H, dd, *J* = 15.0, 5.4 Hz, *Hβ’*), 3.25 (1H, dd, *J* = 15.0, 9.6 Hz, *Hβ”*). ^{13}C NMR (101 MHz, D_2O) δ 171.94 (C=O), 137.99 (C8), 127.02 (C2), 124.74 (C4), 122.92 (C9), 122.81 (C6), 120.17 (C7), 110.97 (C5), 106.82 (C3), 54.81 (Cα), 27.08 (Cβ). HRMS (+ESI) *m/z*: calculated for $C_{11}H_{12}ClN_2O_2$ (M+H): 239.0582. Found 239.0585 (Δ 1.3 ppm).

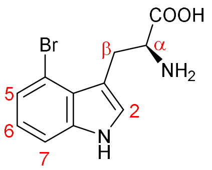

(**14**)

L-4-bromotryptophan (14): ^{1}H NMR (400 MHz, D_2O) δ 7.34 (1H, d, *J* = 8.1 Hz, *H5*), 7.17 (1H, d, *J* = 8.1 Hz, *H7*), 7.16 (1H, s, *H2*), 6.95 (1H, t, *J* = 7.9 Hz, *H6*), 4.36 (1H, dd, *J* = 10.0, 5.3 Hz, *Hα*), 3.70 (1H, dt, *J* = 9.2, 4.6 Hz, *Hβ’*), 3.05 (1H, dd, *J* = 15.1, 10.1 Hz, *Hβ”*). ^{13}C NMR (101 MHz, D_2O) δ 171.72 (*C=O*), 137.75 (*C8*), 127.26 (*C2*), 124.17 (*C4*), 123.52 (*C6*), 122.95 (*C7*), 112.58 (*C9*), 111.42 (*C5*), 107.10 (*C3*), 54.65 (*Cα*), 26.78 (*Cβ*). HRMS (+ESI) *m/z*: calculated for $C_{11}H_{12}BrN_2O_2$ (M+H): 283.0076 (100%), 285.0056 (97.3%). Found 283.0080 (Δ1.4 ppm), 285.0059 (Δ 1 ppm).

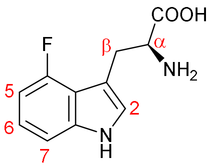

(**15**)

L-4-fluorotryptophan (15): ^{1}H NMR (400 MHz, D_2O) δ 7.21 – 7.10 (2H, m, *H7H2*), 7.04 (1H, dd, *J* = 13.3, 8.0 Hz, *H5*), 6.71 (1H, dd, *J* = 11.6, 7.9 Hz, *H6*), 4.22 (1H, dd, *J* = 8.1, 5.5 Hz, *Hα*), 3.46 (1H, dd, *J* = 15.1, 5.3 Hz, *Hβ*), 3.21 (1H, dd, *J* = 15.0, 8.4 Hz, *Hβ*). ^{13}C NMR (101 MHz, D2O) δ 171.69 (*C=O*), 156.43 (d, J = 242.4 Hz *C4*), 139.23 (d, J = 11.8 Hz *C8*), 125.61 (*C2*), 122.66 (d, J = 7.8 Hz *C7*), 115.01 (d, J = 20.2 Hz *C9*), 108.21 (d, J = 3.2 Hz *C6*), 104.82 (*C3*), 104.25 (d, J = 19.3 Hz *C5*), 53.99 (*Cα*), 27.02 (*Cβ*). HRMS (+ESI) *m/z*: calculated for $C_{11}H_{12}FN_2O_2$ (M+H): 223.0877. Found 223.0879 (Δ 0.7 ppm)

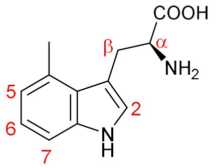

(**16**)

L-4-methyltryptophan (16): ^{1}H NMR (400 MHz, D2O) δ 7.20 (1H, d, J = 8.2 Hz, H5), 7.10 (1H, s, H2), 6.98 (1H, t, J = 7.7 Hz, H6), 6.76 (1H, d, J = 7.1 Hz, H7), 4.09 (1H, dd, J = 10.1, 4.8 Hz, Hα), 3.57 (1H, dd, J = 15.4, 4.8 Hz, Hβ), 3.10 (1H, dd, J = 15.4, 10.2 Hz, Hβ), 2.50 (3H, s, Me). ^{13}C NMR (101 MHz, D2O) δ 171.49 (C=O), 136.93 (C8), 130.34 (C4), 125.55 (C2), 124.57 (C9), 122.34 (C6), 120.97 (C5), 109.92 (C7), 107.57 (C3), 54.50 (Cα), 27.69 (Cβ), 19.20 (Me). (+ESI) *m/z*: calculated for $C_{12}H_{15}N_2O_2$ (M+H): 219.1128. Found 219.1151 (Δ 10 ppm).

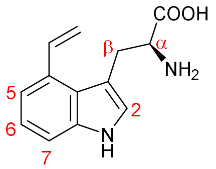

(**17**)

L-4-vinyltryptophan (17): ^{1}H NMR (400 MHz, D2O) δ 7.47 (1H, dd, J = 17.3, 11.0 Hz, HC=), 7.41 (1H, d, J = 8.1 Hz, H5), 7.28 (1H, d, J = 7.2 Hz, H7), 7.25 (1H, s, H2), 7.19 (1H, t, J = 7.7 Hz, H6), 5.77 (1H, dd, J = 17.3, 1.4 Hz, =CH_2), 5.46 (1H, dd, J = 10.9, 1.4 Hz, =CH_2), 3.93 (1H, dd, J = 10.4, 4.6 Hz, Hα), 3.63 (1H, dd, J = 15.3, 4.5 Hz, Hβ), 3.11 (1H, dd, J = 15.4, 10.5 Hz, Hβ). ^{13}C NMR (101 MHz, D2O) δ 174.83 (C=O), 137.32 (C8), 135.05 (HC=), 131.41 (C2), 126.25 (C4), 123.29 (C9), 122.33 (C5), 116.89 (C7), 116.68 (=CH2), 111.62 (C6), 108.51 (C3), 55.90 (Cα), 29.01 (Cβ). HRMS (+ESI) *m/z*: calculated for $C_{13}H_{15}N_2O_2$ (M+H): 231.1128. Found 231.1183 (Δ 23 ppm).

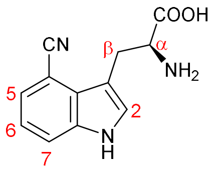

(**18**)

L-4-cyanotryptophan (18): ^{1}H NMR (400 MHz, D2O+100mM DCl) δ 7.64 (1H, d, J = 8.3 Hz, H5), 7.43 (1H, d, J = 7.4 Hz, H7), 7.33 (1H, s, H2), 7.16 (1H, t, J = 7.9 Hz, H6), 4.28 (1H, dd, J = 9.4, 5.8 Hz, Hα), 3.61 (1H, dd, J = 15.5, 5.2 Hz, Hβ), 3.23 (1H, dd, J = 15.3, 9.5 Hz, Hβ). ^{13}C NMR (101 MHz, DMSO) δ 170.32 (C=O), 136.56 (C8), 128.86 (C2), 125.84 (C4)125.58 (C5), 120.97 (C7), 119.29 (C9), 117.18 (C6), 106.73 (C7), 100.05 (C3), 52.53 (C11), 40.06, -38.81 (DMSO), 25.61 (C10). HRMS (+ESI) *m/z*: calculated for $C_{12}H_{12}N_3O_2$ (M+H): 230.0929. Found 230.0925 (Δ1.7 ppm).

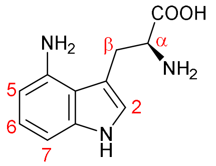

(**19**)

L-4-aminotryptophan (19): ^{1}H NMR (400 MHz, D_2O+100mM DCl) δ 7.45 (1H, d, *J* = 8.3 Hz, H5), 7.30 (1H, s, H2), 7.15 – 7.09 (1H, m, H6), 6.98 (1H, d, *J* = 7.5 Hz, H7), 4.19 – 4.08 (1H, m, Hα), 3.44 (1H, dd, *J* = 16.2, 5.7 Hz, Hβ), 3.32 (1H, dd, *J* = 16.3, 4.5 Hz, Hβ). ^{13}C NMR (101 MHz, D_2O) δ 172.03 (C=O), 137.89 (C8), 127.12 (C2), 121.83 (C7), 121.72 (C6), 121.20 (C4), 119.32 (C9), 113.33 (C5), 104.41 (C3), 53.67 (Cα), 25.95 (Cβ). HRMS (+ESI) *m/z*: calculated for $C_{11}H_{14}N_3O_2$ (M+H): 220.1081. Found 220.1084 (Δ1.4 ppm).

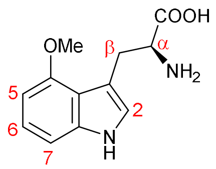

(**20**)

L-4-methoxytryptophan (20): ^{1}H NMR (400 MHz, MeOD) δ 7.05 (2H, dd, J = 10.8, 4.9 Hz, H2 H6), 7.02 – 6.98 (1H, m, H5), 6.56 – 6.48 (1H, m, H7), 4.31 (1H, dd, J = 9.8, 4.3 Hz, Hα), 3.95 (3H, s, OMe), 3.74 (1H, dd, J = 14.6, 4.2 Hz, Hβ), 3.13 (1H, dd, J = 14.6, 9.8 Hz, Hβ). ^{13}C NMR (101 MHz, MeOD) δ 172.45 (C=O), 155.36 (C4), 140.29 (C8), 124.56 (C2), 123.92 (C6), 118.01 (C9), 108.68 (C5), 106.27 (C3), 100.32 (C7), 56.02 (Cα), 55.57 (Me), 29.70 (Cβ). HRMS (+ESI) *m/z*: calculated for $C_{12}H_{15}N_2O_3$ (M+H): 235.1077. Found 235.1081 (Δ 1.7 ppm)

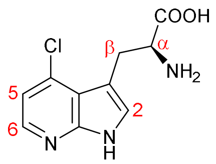

(**21**)

4-Chloro-7-aza tryptophan (21): ^{1}H NMR (400 MHz, D_2O) δ 8.05 (1H, d, *J* = 6.3 Hz, H6), 7.61 (1H, d, *J* = 6.3 Hz, H5), 7.52 (1H, s, H2), 4.26 (1H, dd, *J* = 9.1, 6.2 Hz, Hα), 3.69 (1H, dd, *J* = 15.2, 6.1 Hz, Hβ), 3.24 (1H, dd, *J* = 15.3, 9.2 Hz, Hβ). ^{13}C NMR (101 MHz, D2O) δ 171.4 (C=O), 140.61 (ArC), 134.0 (C2), 133.5 (ArC), 130.00 (C6), 123.1 (ArC), 120.46 (C5), 109.2 (ArC), 54.4 (Cα), 26.1 (Cβ). HRMS (+ESI) *m/z* calculated for $C_{10}H_{11}N_3O_2Cl$ (M+H): 240.0534. Found 240.0545 (Δ 4 ppm).

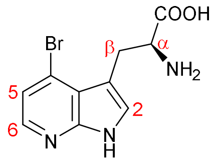

(**22**)

4-Bromo-7-aza tryptophan (22): ^{1}H NMR (400 MHz, D_2O) δ 8.25 (1H, d, *J* = 6.6 Hz, H6, 7.61 (1H, s, H2), 7.53 (1H, d, *J* = 6.6 Hz, H5), 4.34 (1H, dd, *J* = 8.8, 6.2 Hz, Hα), 3.71 (1H, dd, *J* = 15.4, 6.2 Hz, Hβ), 3.36 (1H, dd, *J* = 15.4, 8.8 Hz Hβ). ^{13}C NMR (101 MHz, D2O) δ 170.9 (C=O), 145.1 (ArC), 139.5 (ArC), 133.8 (C6), 130.1 (C2), 122.3 (ArC), 117.3 (C5), 109.5 (ArC), 53.8 (Cα), 26.0 (Cβ). HRMS (+ESI) *m/z* calculated for $C_{10}H_{11}N_3O_2Br$ (M+H): 284.0029**.** Found 284.0028 (Δ 0.4 ppm)

Construction of heterologous txtA-txtB pathway. NRPS gene *txtA* was amplified from *S. scabies* genomic DNA using the following primers designed to enable Gibson assembly of the thaxtomin NRPS genes into pIJ86: 5'-acaatcgtgccggttggtaggaggctctagagATGTCGCACCTGACCGG-3' and 5'-ctcctgggagTCACTGCAGTGG-3'. The second NRPS gene *txtB* was also amplified from the genomic DNA using the following primers: 5'-actgcagtgactcccaggaggctgtcaATGAGGTCCACTCGATGTC-3' and 5'-cggtcgactctagagCTACGGCCGTGGTGAGA-3'. Overlaps for assembly are shown in lower case, gene specific sequences in uppercase and inserted RBS/spacer sequences underlined. PCR products of the correct size were purified by gel electrophoresis and mixed together with pIJ86 digested with BamHI and assembly performed with the Gibson Assembly Master Mix (New England Biolabs) as per manufacturer's instructions to form plasmid pMW224. The mixture was then transformed into *E. coli* DH5α competent cells and the resulting colonies screened for successful assembly by colony PCR. Successful colonies were then picked and plasmid DNA isolated and assembly checked by restriction digest analysis. Successfully assembled pMW224 was transformed into *E. coli* ET12567 (pUZ8002) and conjugated into *S. albus* to form *S. albus::txtAB*.

Construction of heterologous txtA-txtB-txtC pathway. NRPS gene *txtA* was amplified from *S. scabies* genomic DNA as above. *TxtB* was amplified from the genomic DNA using the following primers: 5’-actgcagtgactcccaggaggctgtcaATGAGGTCCACTCGATGTC-3’ and 5’-ctcctggtgtCTACGG-CCGTGGTGAGA-3’.TxtC was amplified from genomic DNA with the primers: 5’-acggccgtag-acaccaggaggtcgtacATGGAATCTCCGGCCAC-3’ and 5’-catgccggtcgactctagagTCACCAGGTCATG-GGCA-3’. Overlaps for assembly are shown in lower case, gene specific sequences in uppercase and inserted RBS/spacer sequences underlined. PCR products of the correct size were purified by gel electrophoresis and mixed together with pIJ86 digested with BamHI and assembly performed with the Gibson Assembly Master Mix as per manufacturer’s instructions to form plasmid pMW233. The mixture was then transformed into *E. coli* DH5α competent cells and the resulting colonies screened for successful assembly by colony PCR. Successful colonies were then picked and plasmid DNA isolated and assembly checked by restriction digest analysis. Successfully assembled pMW233 was transformed into *E. coli* ET12567 (pUZ8002) and conjugated into *S. albus* to form *S. albus::txtABC*.

Construction of heterologous txtA-txtB-trpA-trpB pathway. NRPS gene txtA was amplified from *S. scabies* genomic DNA with the primers 5’-acaatcgtgccggttggtaggaggctctagagATGTCGCAC-CTGACCGGT-3’ and 5’-acagcctcctgggagTCACTGCAGTGGGAGGTC-3’. TxtB was amplified from the genomic DNA using the following primers: 5’-actgcagtgactcccaggaggctgtcaATGAGGTCCACTCGA-TGTC-3’ and 5’-tgttagagcccgcacaCTACGGCCGTGGTGAGAA-3’. Tryptophan synthase genes were codon optimised for *Streptomyces coelicolor* codon usage and ordered from GeneArt (Invitrogen). The following primers were used to prepare trpA for assembly: 5’-acggccgtagtgtgcgggc-tctaacacgtcctagtatggtaggatgagcaatcgagcaaaggaggtacggacATGGAGCGCTACGAGAACCTGTTCG-3’ and 5’-ttccctcctggacggTTAGGCGCGGGAGGCGGC-3’. TrpB was amplified using the following primers: 5’-ccgcgcctaaccgtccaggagggaaattATGACCACCCTGCTGAACCC-3’ and 5’-catgccggtcga-ctctagagTTAGATCTCGCCGCGGGC-3’. Overlaps for assembly are shown in lower case, gene specific sequences in uppercase and inserted RBS/spacer sequences underlined. PCR products of the correct size were purified by gel electrophoresis and mixed together with pIJ86 digested with BamHI and assembly performed with the Hifi Assembly Master Mix (New England Biolabs) as per manufacturer’s instructions to form plasmid pMW236. The mixture was then transformed into *E. coli* DH5a competent cells and the resulting colonies screened for successful assembly by colony PCR. Successful colonies were then picked and plasmid DNA isolated and assembly checked by restriction digest analysis. Successfully assembled pMW236 was transformed into *E. coli* ET12567 (pUZ8002) and conjugated into *S. albus* to form *S. albus::txtABtrpAB*.

Generation of Thaxtomin Analogues. 10 mL of tryptone soya broth (TSB, Oxoid) was inoculated with 20 µL of a *S. albus* spore stock (either *S. albus::txtAB*, *S. albus::txtABC*, or *S. albus::txtABtrpAB*) and grown for 48 hours at 28 °C in a 25 mL conical flask fitted with a spring. 1.5 mL of this seed culture was then used to inoculate 150 mL of fermentation medium (18 g L^{-1} malt extract, 7 g L^{-1} peptone, 5 g L^{-1} NaCl, pH 8.5). The culture was incubated for 3 days, or until evidence of strong growth was seen. Cultures were then supplemented with either L-tryptophan analogues or indole analogues as appropriate (50 µM). Growth was continued for 5-7 days. Centrifugation (4°C, 30 min, 10,000 x g) removed the cells and the supernatant was extracted with EtOAc (3 x 150 mL) and then reduced to dryness. The residue was re-dissolved in 10 mL of MeOH and analysed by LCMS on an Agilent 1200 series HPLC fitted with a Phenomenex C18 Kinetex column (100 mm x 2.1 mm, 2.6µm) using a flow rate of 0.2 mL min^{-1} and the following gradient: 0-3 mins 5% B; 3-8 mins 5-70% B; 8-9 mins 70-95% B; 9-13 mins 95% B; 13-14 mins 95-5% B; 14-20 mins 5% B where A was 0.1% formic acid in water and B was 0.1% formic acid in ACN. Eluent from the HPLC was analysed by a 6510 QTof mass spectrometer (Agilent). Fragmentation analysis was carried out at 175V (CID@15). Production of the new analogues was assessed by peak area integration relative to cultures fed with the natural substrate 4-nitrotryptophan and titres estimated with a standard curve of thaxtomin A (Figure S1). High resolution LCMS was carried out on a Thermo Ultimate 3000 HPLC fitted with a Thermo Accucore C18 column (100 mm x 2.1 mm, 2.6 µm) using a flow rate of 0.3 mL min^{-1} and the following gradient: 0-2 mins 5% B; 2-10 mins 5-85% B; 10-10.5 mins 85-95% B; 10.5-12.5 mins 95% B; 12.5-13 mins 95-5% B; 13-15 mins 5% B where A was 0.1% formic acid in water and B was 0.1% formic acid in MeOH. Eluent from the HPLC was analysed by a Q Exactive Plus mass spectrometer (Thermo).

5L preparative purification of thaxtomin analogues. Multiple replicate cultures were set up as above to a total volume of 5L of media. The flasks were grown at 30 °C for 2 days with 150 rpm agitation, or until strong evidence of growth was seen. A total amount of 500 mg of L-tryptophan analogues (4-nitro- or 4-bromo-) was evenly distributed amongst the flasks with feeding spread equally over 5 days. On day 7 of fermentation the cell biomass was removed by centrifugation (4°C, 30 min, 6000 g), the supernatants combined and the volume reduced *in vacuo* to around 800 ml. This was then extracted with EtOAc (4 x 800 ml) and then reduced to dryness. The residue was re-dissolved in 10 ml of MeOH and purified by RP-HPLC on a Phenomenex Gemini C18 column (5 μM, 110Å, 250 x 10 mm) at 5 ml min^{-1} with a gradient of H_2O + 0.1% FA versus (B) ACN 0.1% FA (0-5 min 5% B, 5-26 min 5-95% B curve -1, 26-32 min 95% B, 32-33 min 95-5% B, 33-38 min 5% B). The thaxtomin containing fractions were combined and then reduced in volume to 3 ml and further purified using an isocratic gradient on the Phenomenex Gemini C18 column (5 μM, 110Å, 250 x 10 mm) at 5 ml min^{-1} with a gradient of H_2O + 0.1% FA versus (B) ACN 0.1% FA (0-60 min 50% B, 60-61 min 50-95% B, 61-70 min 95% B, 70-71 min 95-50% B, 71-80 min 50% B) to yield thaxtomin analogues:

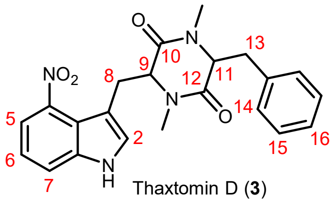

Thaxtomin D (**3**)

Thaxtomin D (3): ^{1}H NMR (400 MHz, MeOD) 7.85 (1 H, dd, *J* 8.0, 1.0 Hz, H5), 7.73 (1 H, dd, *J* 8.0, 1.0 Hz, H7), 7.41 – 7.33 (2 H, m, Ph), 7.28 (1 H, d, *J* 7.4 Hz, Ph), 7.23 (1 H, t, *J* 8.0 Hz, H6), 7.14 – 7.06 (4 H, m, Ph, H2), 4.18 (1 H, t, *J* 5.2 Hz, H9), 3.97 (1 H, dd, *J* 7.6, 5.6 Hz, H11), 2.98 (1 H, t, *J* 5.2 H13), 2.95 (1 H, t, *J* 5.4 H8), 2.86 (3 H, s, NMe), 2.76 (3 H, s, NMe), 2.62 (1 H, dd, *J* 14.2, 5.2 Hz, H13'), 2.42 (1 H, dd, *J* 14.6, 7.6 Hz, H8'). HRMS (+ESI) *m/z*: calculated for $C_{22}H_{23}N_4O_4$ (M+H): 407.1714. Found 407.1713 (Δ 0.17 ppm).

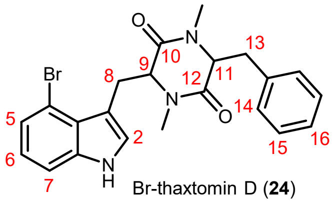

Br-thaxtomin D (**24**)

4-Bromothaxtomin D (24): δ H (800 MHz, Methanol-*d*4) 7.40 (2 H, t, *J* 7.6, H15), 7.33 (1 H, d, *J* 8.0, H5), 7.30 (1 H, t, *J* 7.6, H7), 7.19 (1 H, d, *J* 7.6, H16), 7.16 (2 H, d, *J* 8.0, H14), 6.96 (1 H, t, *J* 7.8, H6), 6.81 (1 H, s, H2), 4.34 (1 H, dd, *J* 8.8, 5.0, H9), 4.29 (1 H, t, *J* 5.0, H11), 3.15 (1 H, dd, *J* 14.3, 5.0, H13), 2.98 (1 H, dd, *J* 13.4, 3.9, H8), 2.96 (1 H, dd, *J* 12.9, 3.9, H13'), 2.92 (3 H, s, NMe), 2.53 (3 H, s, NMe), 2.18 (1 H, dd, *J* 14.7, 8.7, H8'). δ C (201 MHz, Methanol-*d*4) 168.0 (C=O), 167.8 (C=O), 139.5 (ArC), 137.7 (ArC), 131.2 (C14), 130.1 (C15) , 128.6 (C7), 128.2 (C2), 126.3 (ArC), 124.5 (C16), 123.5 (C6), 114.3 (ArC), 112.2 (C5), 111.3 (ArC), 65.5 (C9), 65.2 (C11), 38.6 (C13), 35.1 (NMe), 33.5 (NMe), 31.9 (C8). HRMS (+ESI) *m/z*: calculated for $C_{22}H_{23}N_3O_2{}^{79}Br$ (M+H): 440.0968. Found 440.0972 (Δ 0.8 ppm).

Supplementary Tables and Figures

Table S1. HRMS and relative production/titres of thaxtomin D derivatives produced from supplementing *S. albus::txtAB* (strain i) with modified tryptophan precursors. High resolution MS analysis of produced thaxtomin analogues compared to predicted monoisotopic masses.

Thaxtomin D analogue	Calculated $[M+H]^+$	Observed $[M+H]^+$	Production relative to thaxtomin D
Thaxtomin D (**3**)	407.1714	407.1713<br>(Δ 0.17 ppm)	100%<br>(0.7 mg L^{-1})
Cl (**23**)	396.1473	396.1475<br>(Δ 0.6 ppm)	53%<br>(0.32 mg L^{-1})
Br (**24**)	440.0968	440.0972<br>(Δ 0.8 ppm)	27%<br>(0.17 mg L^{-1})
F (**25**)	380.1769	380.1767<br>(Δ 0.5 ppm)	21%<br>(0.13 mg L^{-1})
Me (**26**)	376.2020	376. 2019<br>(Δ 0.14 ppm)	68%<br>(0.42 mg L^{-1})
Des-nitro (**27**)	362.1863	362.1866<br>(Δ 0.3 ppm)	1-10%

Table S2. High resolution MS analysis (M+H) of hydroxylated thaxtomin analogues produced by *S. albus::txtABC*. When multiple peaks of the same *m/z* are present within a chromatogram (**Figure S4**) they are listed in ascending order of retention time, with earliest eluting peak at the top of the list. Titres estimated by peak area versus standard curve of Thaxtomin A (**Figure S1**).

**Thaxtomin Substituent**		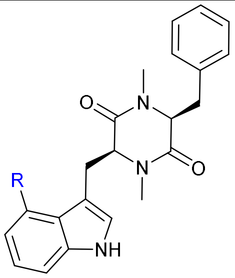	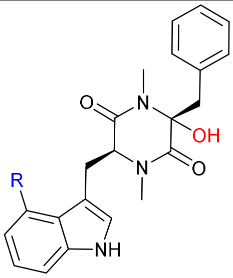	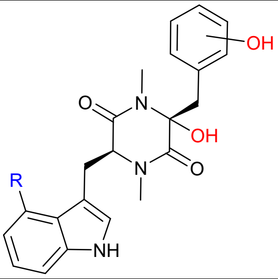	**Estimated titre of main peak (highlighted with *)**
R = NO_2	(Expected)	(407.1714)	(423.1663)	(439.1612)	
	**Found**	**-**	**423.1667 (Δ 0.4 ppm)**	**439.1623 (Δ 1 ppm)<br>439.1616 (Δ 0.3 ppm)*<br>439.1612 (Δ 0.8 ppm)**	**0.05 mg L^{-1}**
R = Cl	(Expected)	(396.1473)	(412.1422)	(428.1372)	
	**Found**	**396.1481 (Δ 0.8 ppm)**	**412.1426 (Δ 0.3 ppm)**	**428.1377 (Δ 0.5 ppm)*<br>428.1377 (Δ 0.5 ppm)<br>428.1378 (Δ 0.6 ppm)**	**0.06 mg L^{-1}**
R = Br	(Expected)	(440.0968)	(456.0917)	(472.0866)	
	**Found**	**-**	**456.0921 (Δ 0.3 ppm)**	**472.0875 (Δ 0.8 ppm)*<br>472.0875 (Δ 0.8 ppm)<br>472.0873 (Δ 0.6 ppm)**	**0.05 mg L^{-1}**
R = F	(Expected)	(380.1769)	(396.1718)	(412.1667)	
	**Found**	**-**	**396.1709 (Δ 2.2 ppm)*<br>396.1715 (Δ 0.8 ppm)**	**412.1655 (Δ 2.9 ppm)**	**0.03 mg L^{-1}**
R = Me	(Expected)	(376.2020)	(392.1969)	(408.1918)	
	**Found**	**376.2023 (Δ 0.3 ppm)**	**392.1972 (Δ 0.4 ppm)***	**408.1921 (Δ 0.3 ppm)<br>408.1924 (Δ 0.6 ppm)**	**0.10 mg L^{-1}**
R = H (desnitro)	(Expected)	(362.1863)	(378.1812)	(394.1761)	
	**Found**	**362.1865 (Δ 0.2 ppm)**	**378.1815 (Δ 0.3 ppm)**	**392.1765 (Δ 0.4 ppm)<br>392.1765 (Δ 0.4 ppm)<br>394.1763 (Δ 0.2 ppm)**	**-**

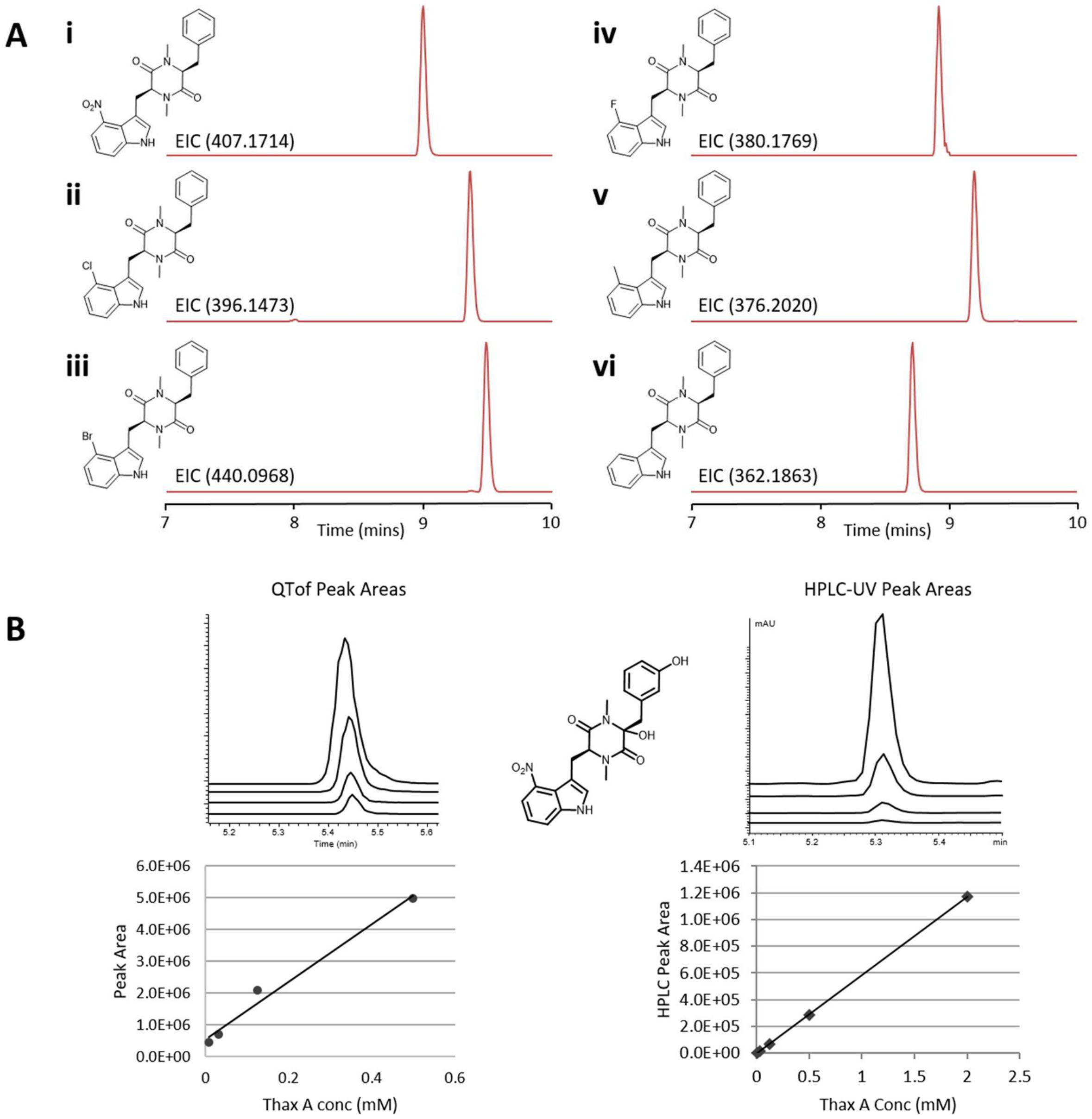

Figure S1. (A) Extracted Ion Chromatograms (EIC) for expected masses (M+H) of thaxtomin D analogues produced by *S. albus::txtAB*. Chromatograms of thaxtomin analogues resulting from *S. albus::txtAB* fed with (**i**) 4-nitrotryptophan; (**ii**) 4-chlorotryptophan; (**iii**) 4-bromotryptophan; (**iv**) 4-fluorotryptophan; (**v**) 4-methyltryptophan; (**vi**) background incorporation of unmodified tryptophan even in non-fed cultures. Accurate mass data for each peak can be found in **table S1**. **(B) Comparison of Standard Curves of Known Thaxtomin A Concentrations Assessed by LCMS and HPLC.** LCMS standard curve of thaxtomin A was compared to a UV standard curve produced by HPLC DAD. Both were in agreement when calculating the concentration of thaxtomin A in samples of known concentration. Validated LCMS standard curve was then used to estimate titres of thaxtomin analogues.

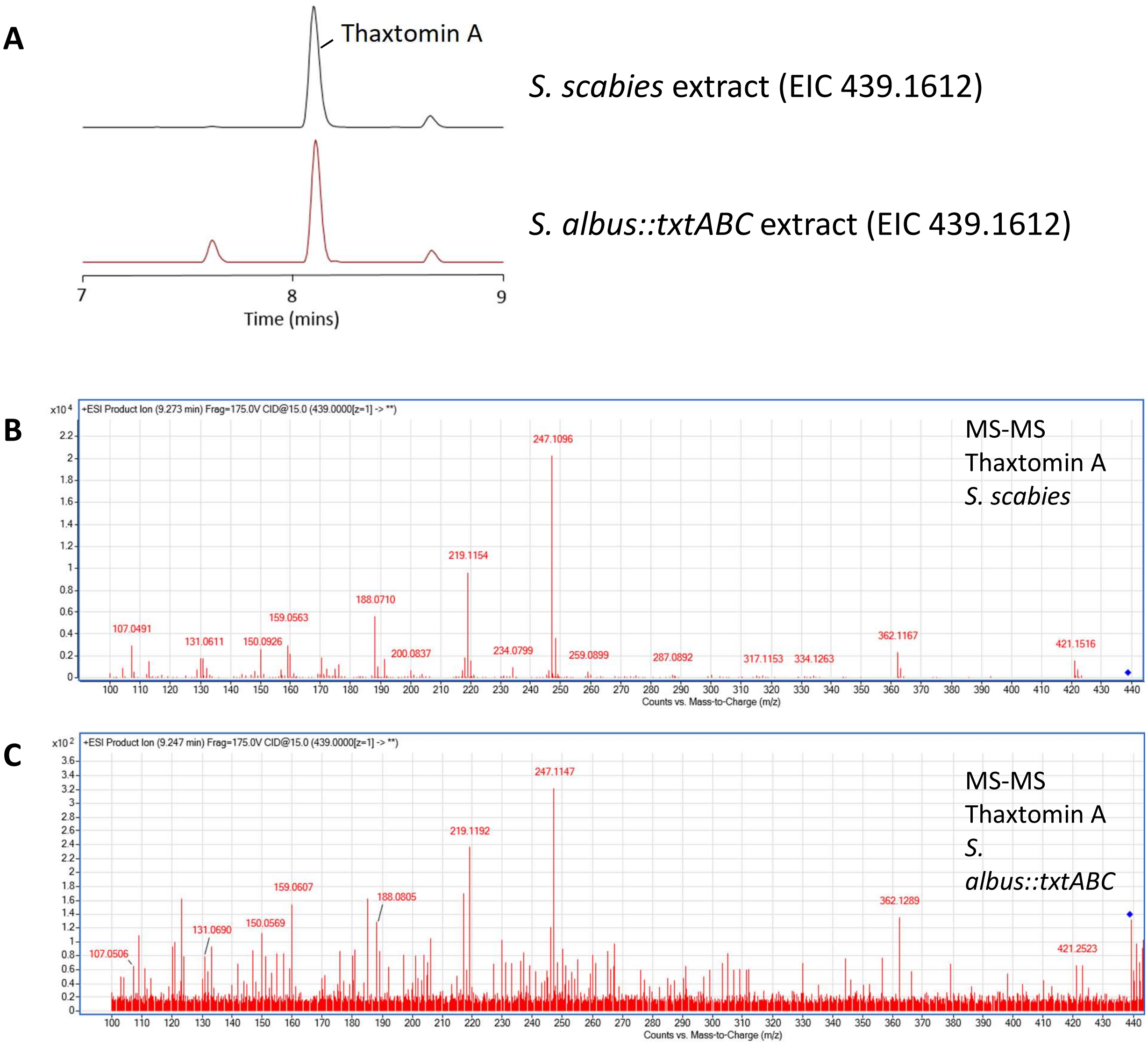

Figure S2. LCMS Comparison between thaxtomin isolated from wild-type *S. scabies* and the *S. albus::txtABC* heterologous host fed with 4-nitrotryptophan. (A) *S. albus::txtABC* produces three di-hydroxylated thaxtomin analogues, *m/z* 439.1612 (M+H). The retention times and *m/z* match with the 3-regioisomers (**5-7**, **Figure 1**) produced by *S. scabies*. The major compound produced by *S. scabies*, Thaxtomin A, is the same major product of the synthetic pathway. MS fragmentation of the major product of the *S. albus::txtABC* pathway (**C**) matches that of thaxtomin A produced by the wild type organism (**B**).

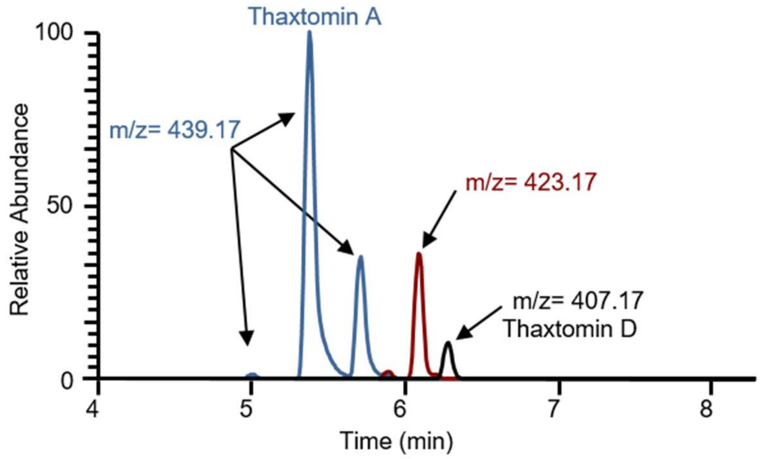

Figure S3. HPLC analysis of hydroxylated thaxtomins from *S. albus::txtABC* (strain ii) cultures fed with 4-NO_2-tryptophan. The HPLC shows extracted ion chromatograms for dihydroxylated thaxtomin (*m/z* 439.17, blue trace), mono-hydroxylated thaxtomin (*m/z* 423.17, red trace) and non-hydroxylated starting material (*m/z* 407.17, black trace). The major product of the pathway is thaxtomin A (RT 5.4 mins)

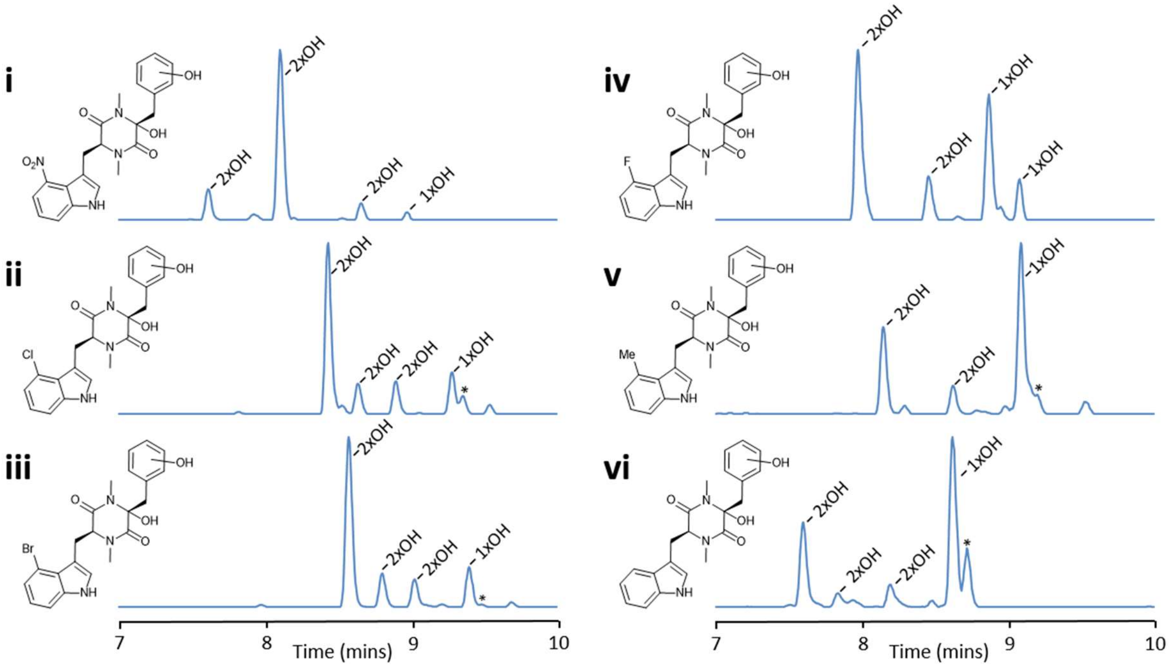

Figure S4. LCMS EICs for expected masses (M+H) of non-hydroxylated, mono-hydroxylated, and di-hydroxylated thaxtomin analogues produced by *S. albus::txtABC* cultures fed with (**i**) **4-nitrotryptophan** *(EIC 407.1714 (thaxtomin D), 423.1663 (monohydroxylated), 439.1612 (dihydroxylated)*; (**ii**) **4-chlorotryptophan** *(EIC 396.1473 (chloro-thaxtomin D), 412.1422 (monohydroxylated), 428.1372 (dihydroxylated)*; (**iii**) **4-bromotryptophan** *(EIC 440.0968 (bromo-thaxtomin D), 456.0917 (monohydroxylated), 472.0866 (dihydroxylated)*; (**iv**) **4-fluorotryptophan** *(EIC 380.1769 (fluoro-thaxtomin D), 396.1718 (monohydroxylated), 412.1667 (dihydroxylated)*; (**v**) **4-methyltryptophan** *(EIC 376.2020 (methyl-thaxtomin D), 392.1969 (monohydroxylated), 408.1918 (dihydroxylated)*; (**vi**) background incorporation of unmodified tryptophan even in non-fed cultures *(EIC 362.1863 (desnitro-thaxtomin D), 378.1812 (monohydroxylated), 394.1761 (dihydroxylated)*. Nitro, bromo and chloro thaxtomin D analogues are converted to mainly a single di-hydroxylated product. Fluoro, methyl and desnitro thaxtomin D result in a mixture of di-hydroxylated thaxtomin and a mono-hydroxylated intermediate. Any remaining starting material is highlighted with an asterisk. Found masses for each peak can be found in **Table S2**.

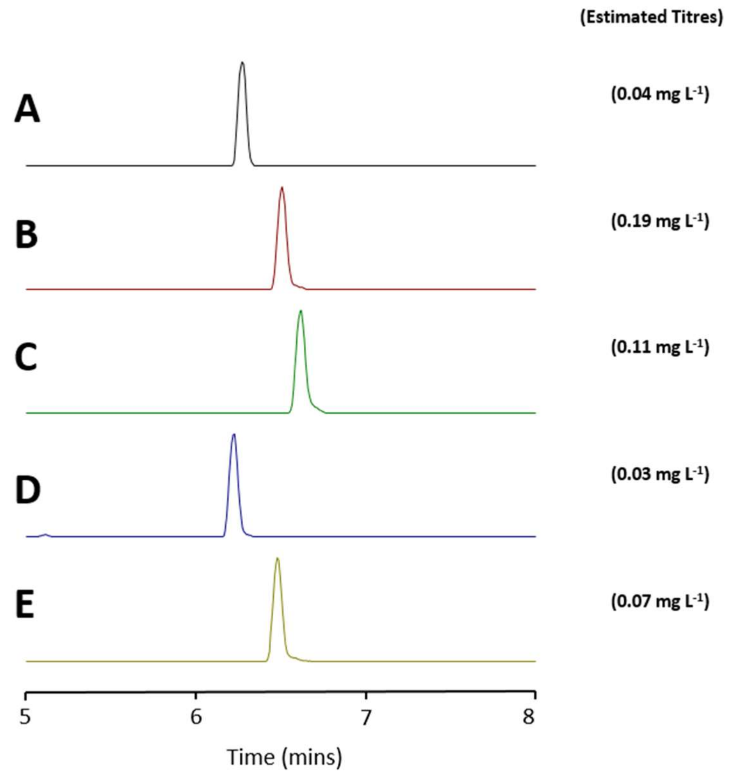

Figure S5. LCMS extracted ion chromatograms for expected masses (M+H) of thaxtomin analogues produced by cultures of *S. albus::txtAB,trpAB* highlighting the formation of (A) Thaxtomin D (*m/z* 407); (B) 4-chloro-thaxtomin D (*m/z* 396); (C) 4-bromo-thaxtomin D (*m/z* 440); (D) 4-fluoro-thaxtomin D (*m/z* 380); and (E) 4-methyl-thaxtomin D (*m/z* 376) when exogenously fed with the appropriate indole analogue. Titres are estimated based on peak areas versus calibration curve of thaxtomin A (**Figure S1**).

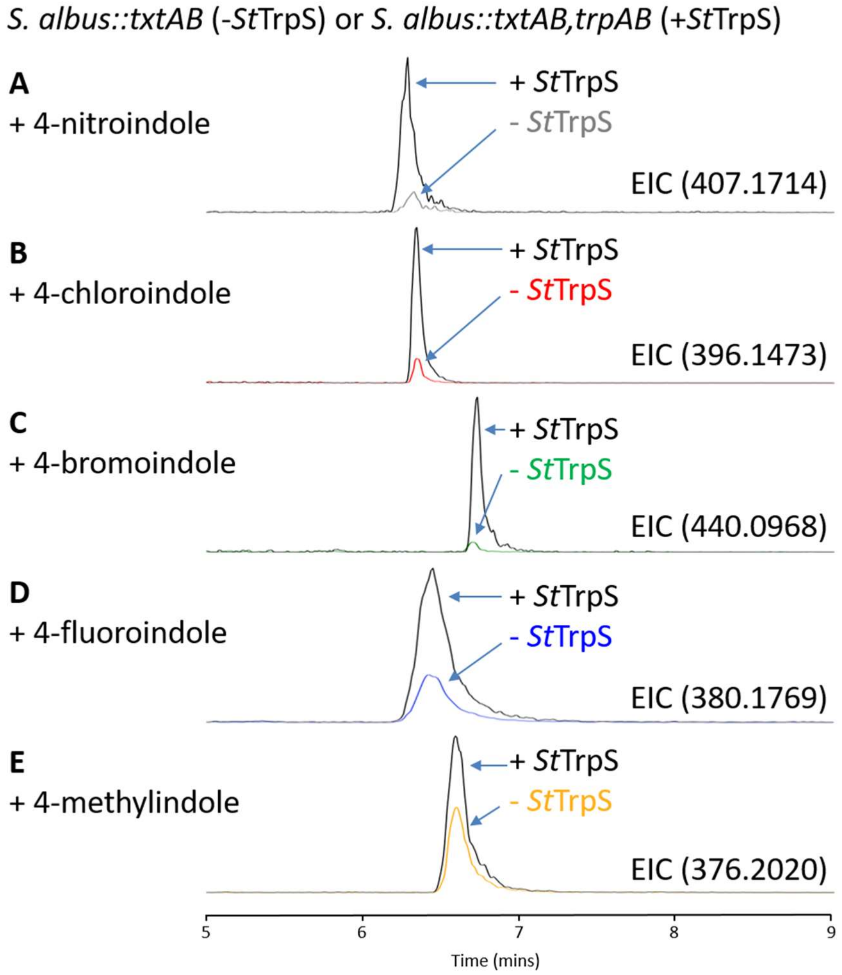

Figure S6. Comparison of LCMS extracted ion chromatograms for expected masses (M+H) of thaxtomin analogues produced by cultures of *S. albus::txtAB,trpAB* (+*St*TrpS) and *S. albus::txtAB* (-*St*TrpS) when exogenously fed with the appropriate indole analogue highlighting the formation of (A) Thaxtomin D (*m/z* 407); (B) 4-chloro-thaxtomin D (*m/z* 396); (C) 4-bromo-thaxtomin D (*m/z* 440); (D) 4-fluoro-thaxtomin D (*m/z* 380); and (E) 4-methyl-thaxtomin D (*m/z* 376). While activity of the *S. albus* natural TrpS can lead to small amounts of thaxtomin product (coloured traces), the yields are greatly increased when the strain is supplemented with the heterologous *St*TrpS from *Salmonella* (black traces).

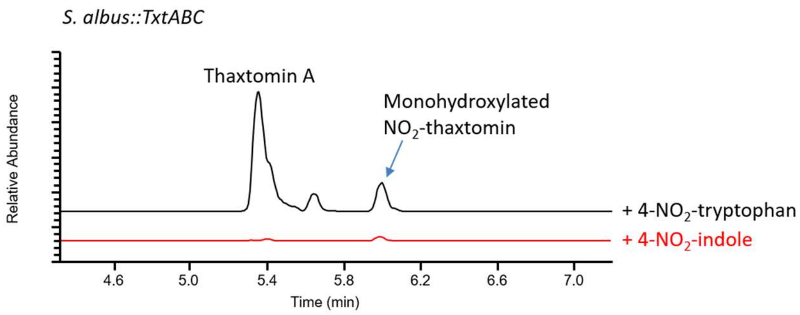

Figure S7. Comparison of LCMS extracted ion chromatograms for expected masses (M+H) of hydroxylated thaxtomin analogues produced by cultures of *S. albus::txtABC* when exogenously fed with 4-nitrotryptophan or 4-nitroindole. Without the heterologous *St*TrpS, the native TrpS of *S. albus* only produces trace amounts of a monohydroxylated thaxtomin analogue. Combined extracted ion chromatograms for dihydroxylated thaxtomin (*m/z* 439.17), mono-hydroxylated thaxtomin (*m/z* 423.17) and non-hydroxylated starting material (*m/z* 407.17).

NMR Spectra

^{1}H NMR

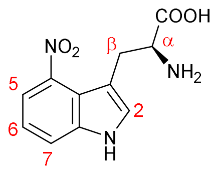

(**12**)

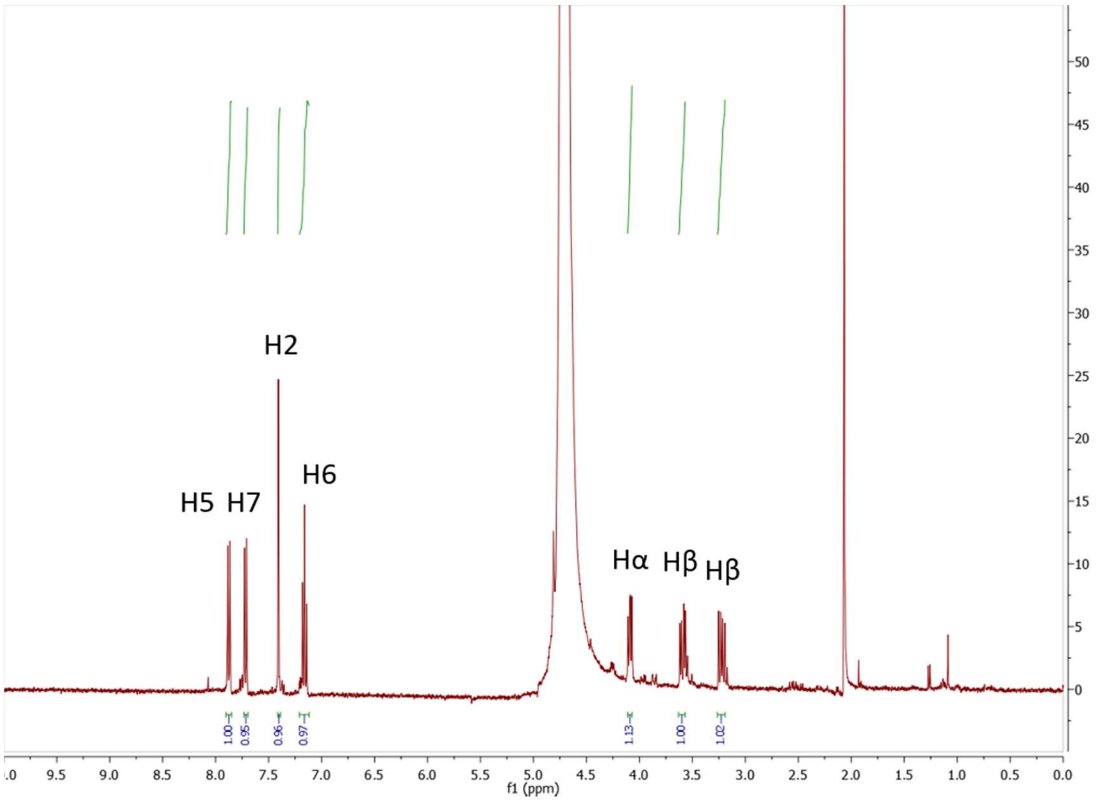

^{13}C NMR

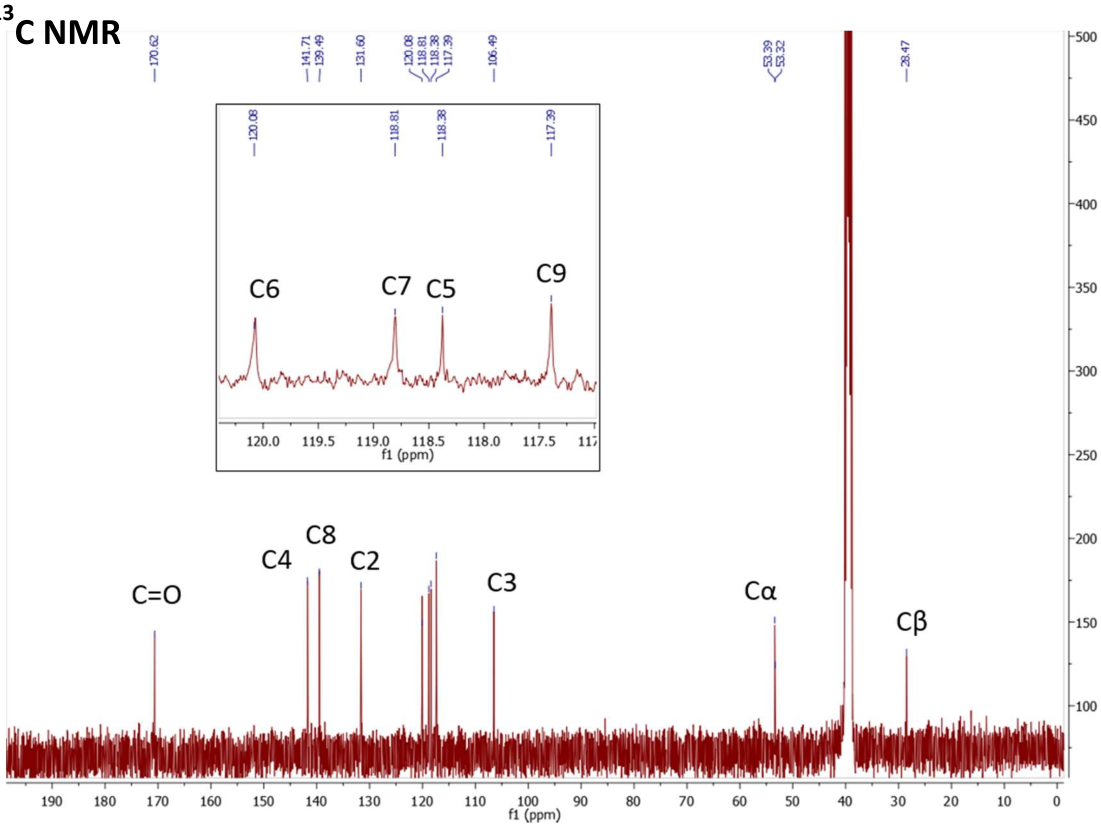

^{1}H NMR

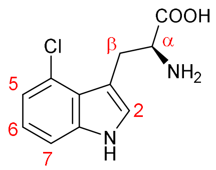

(**13**)

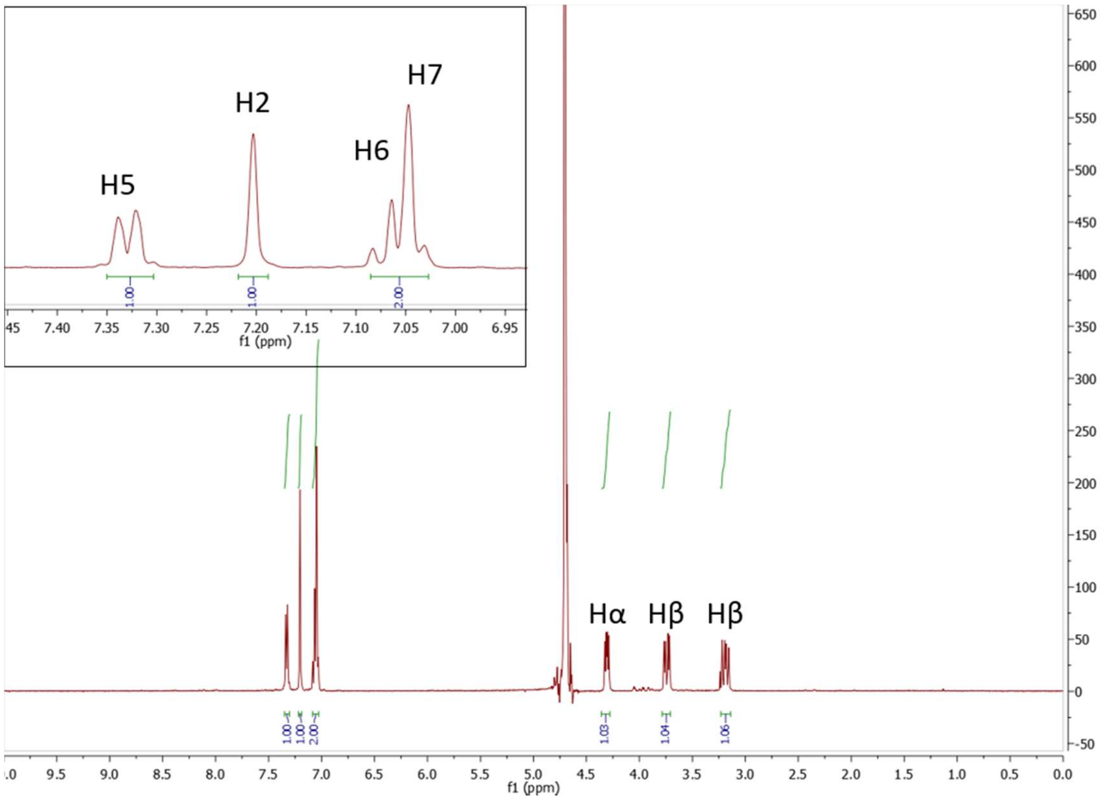

^{13}C NMR

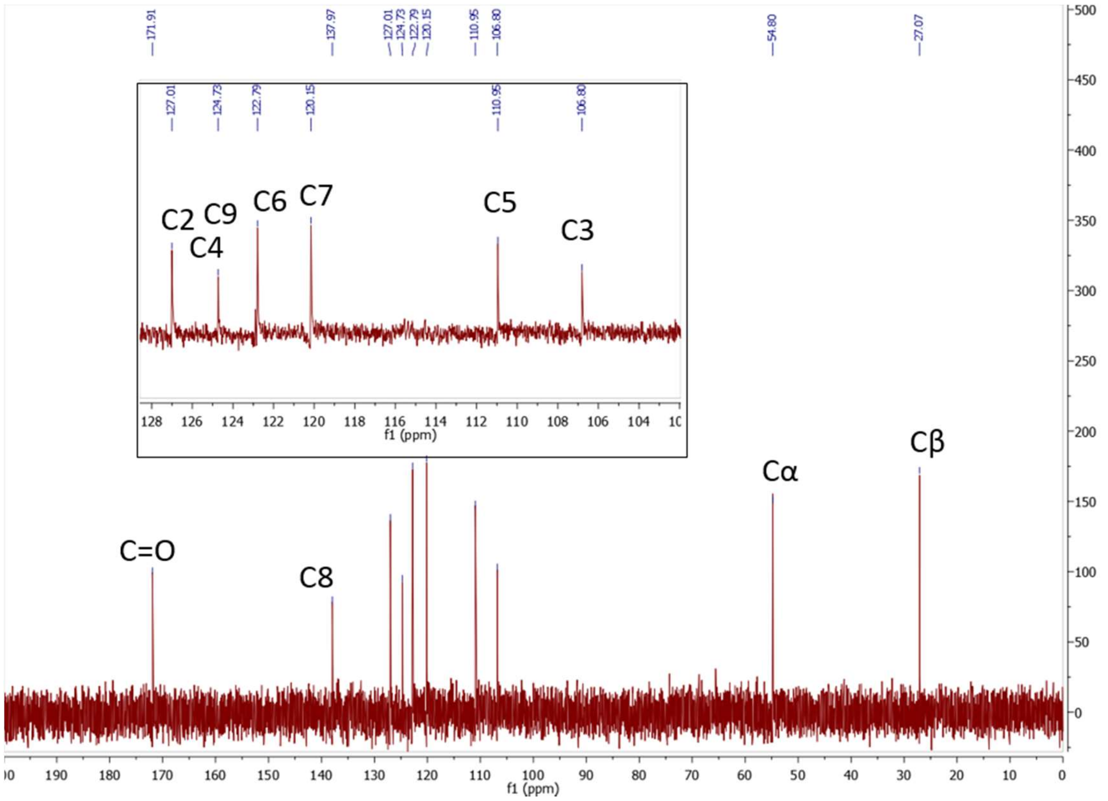

^{1}H NMR

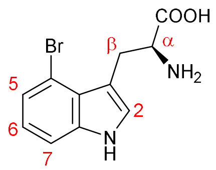

(**14**)

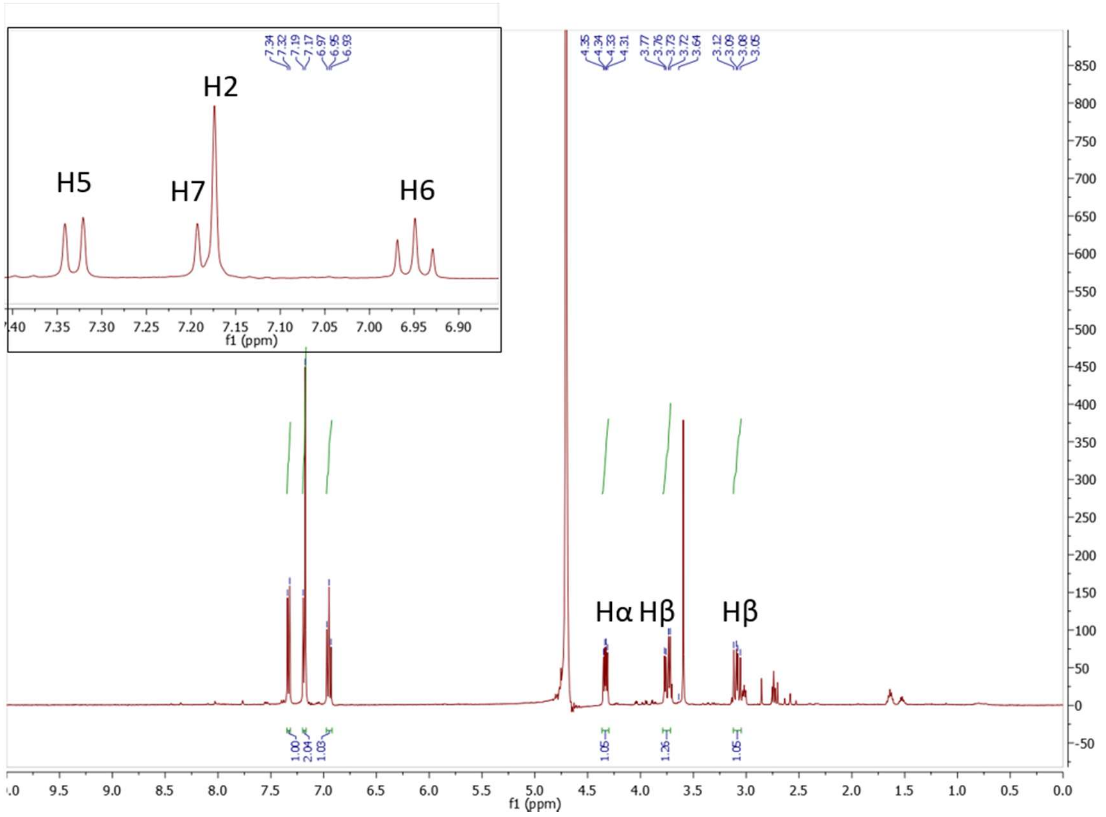

^{13}C NMR

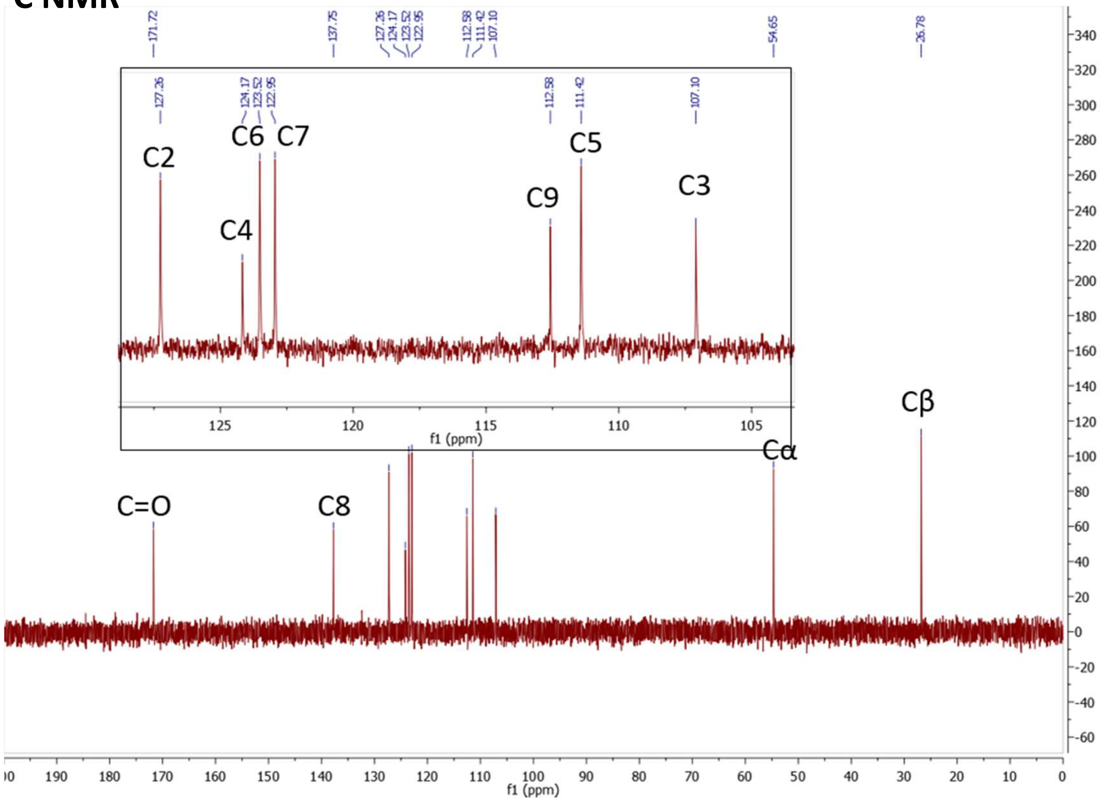

^{1}H NMR

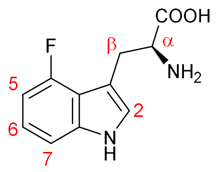

(**15**)

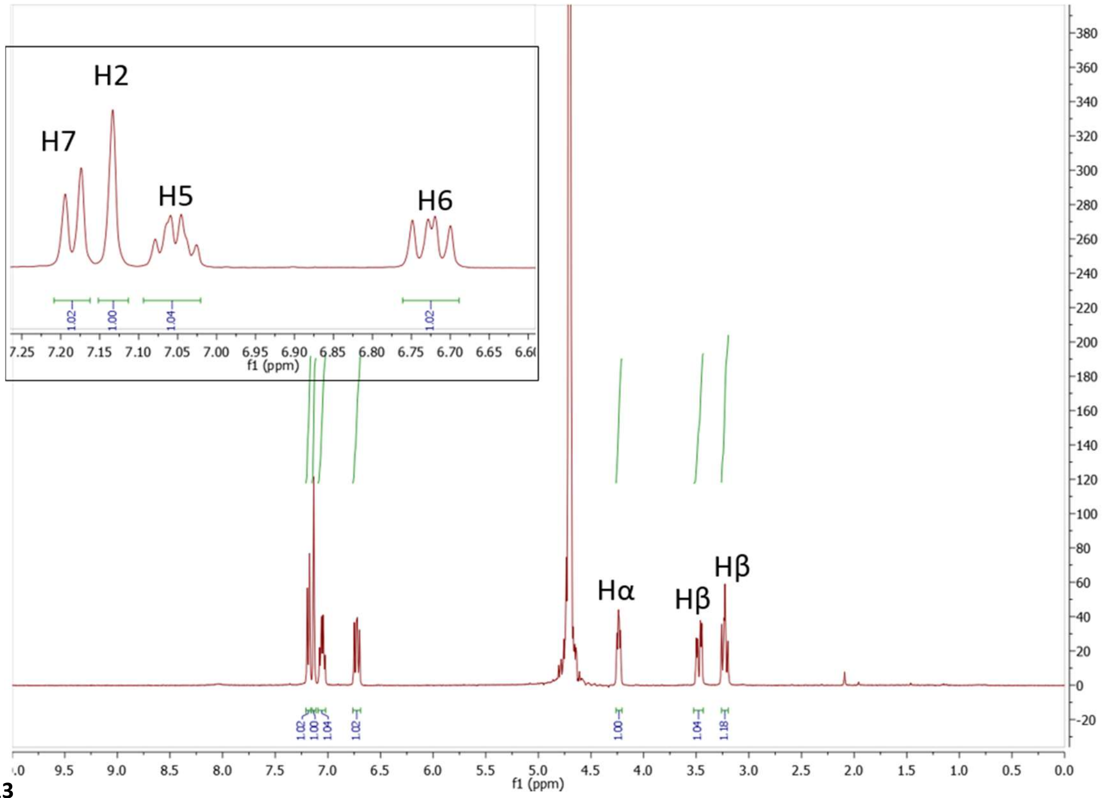

^{13}C NMR

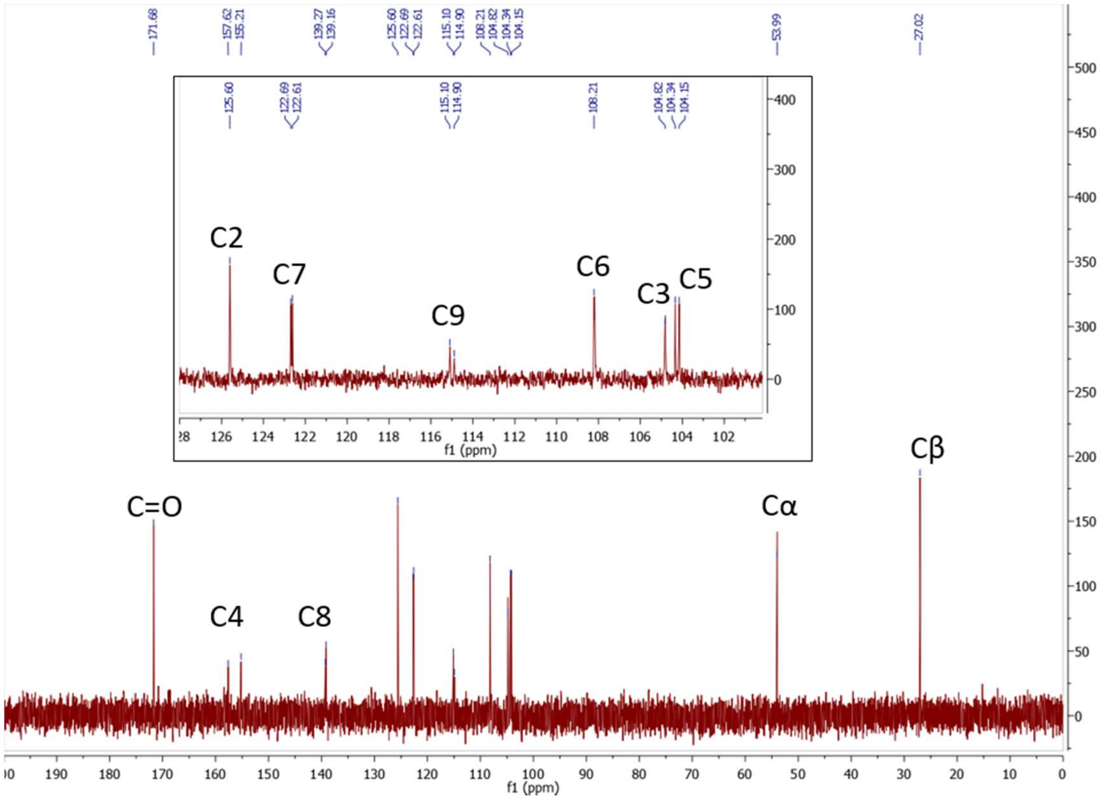

^{1}H NMR

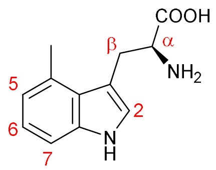

(**16**)

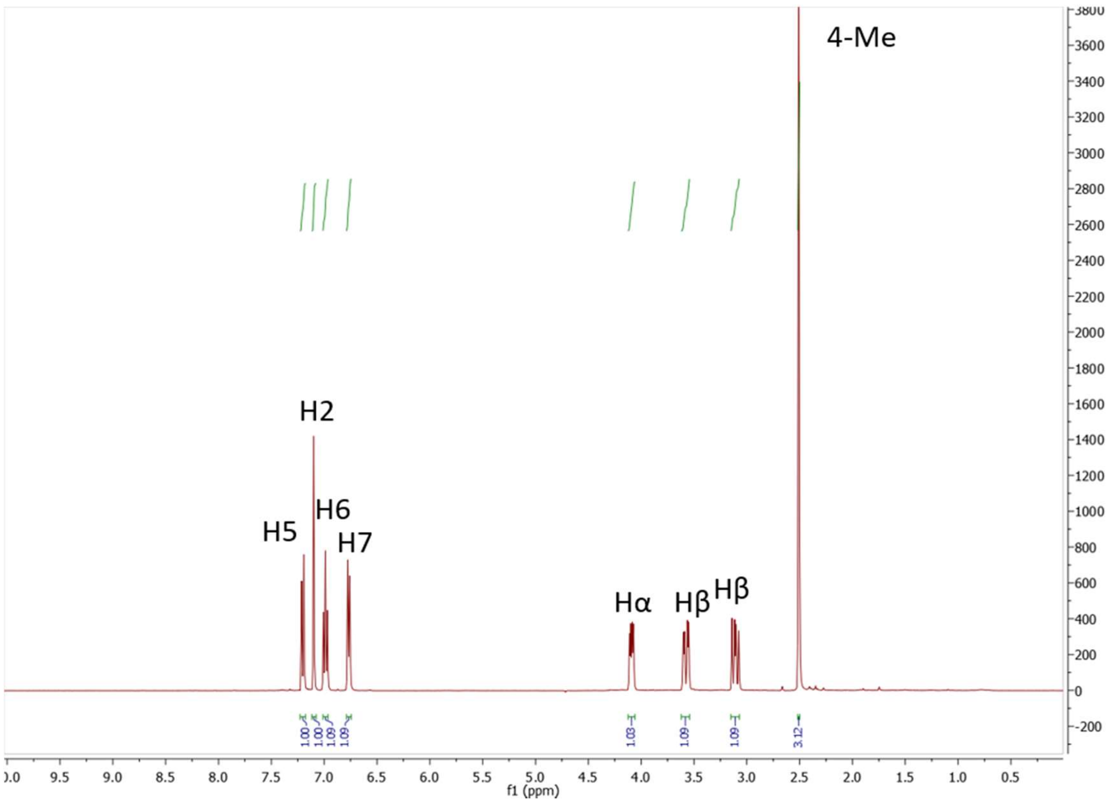

^{13}C NMR

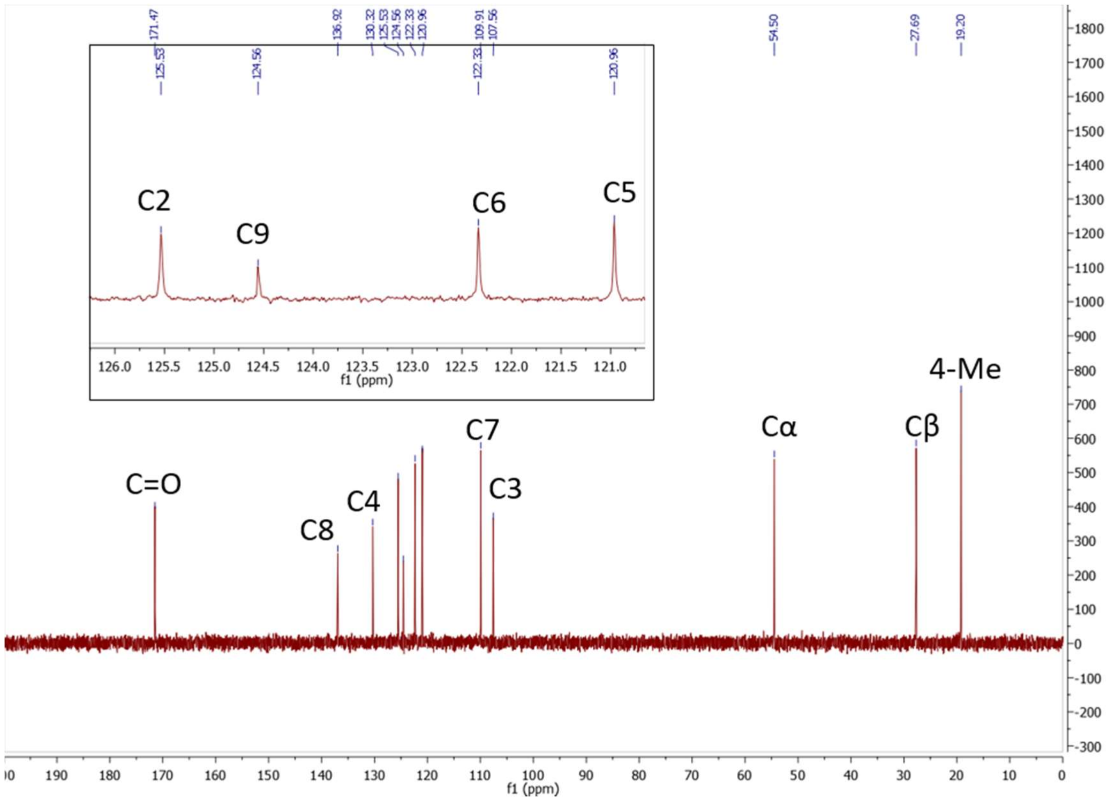

^{1}H NMR

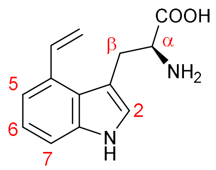

(**17**)

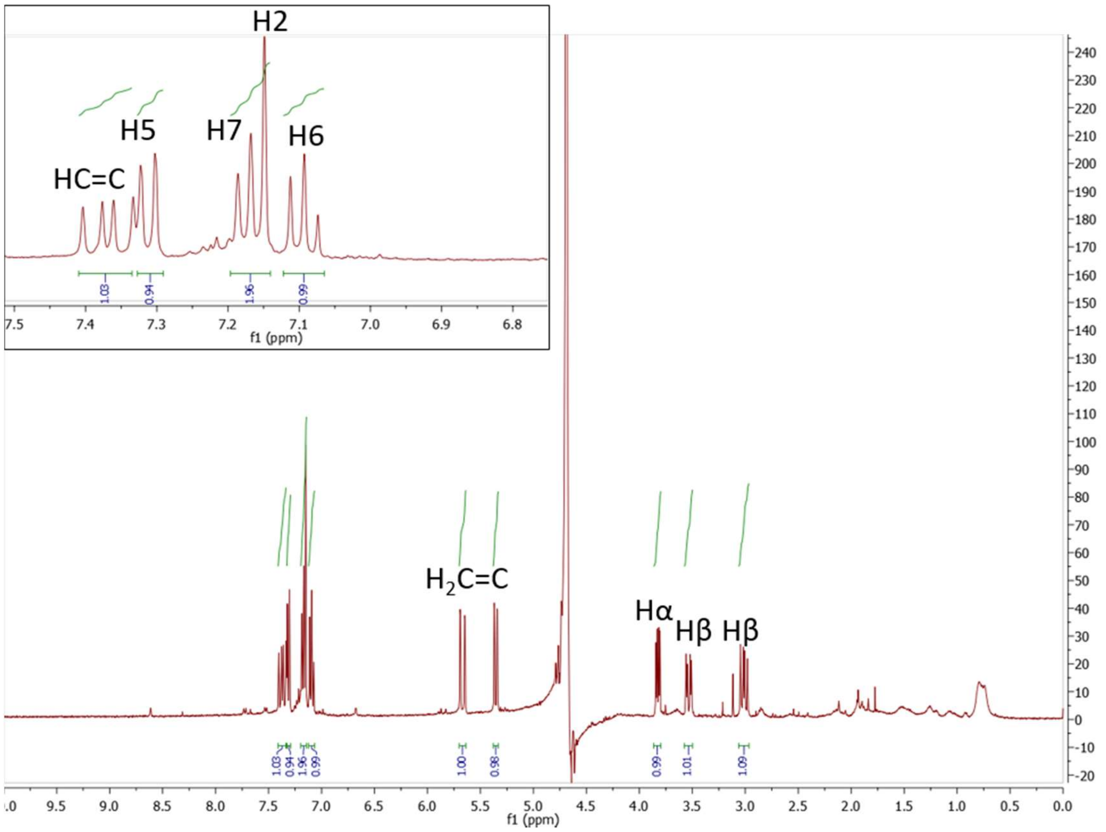

^{13}C NMR

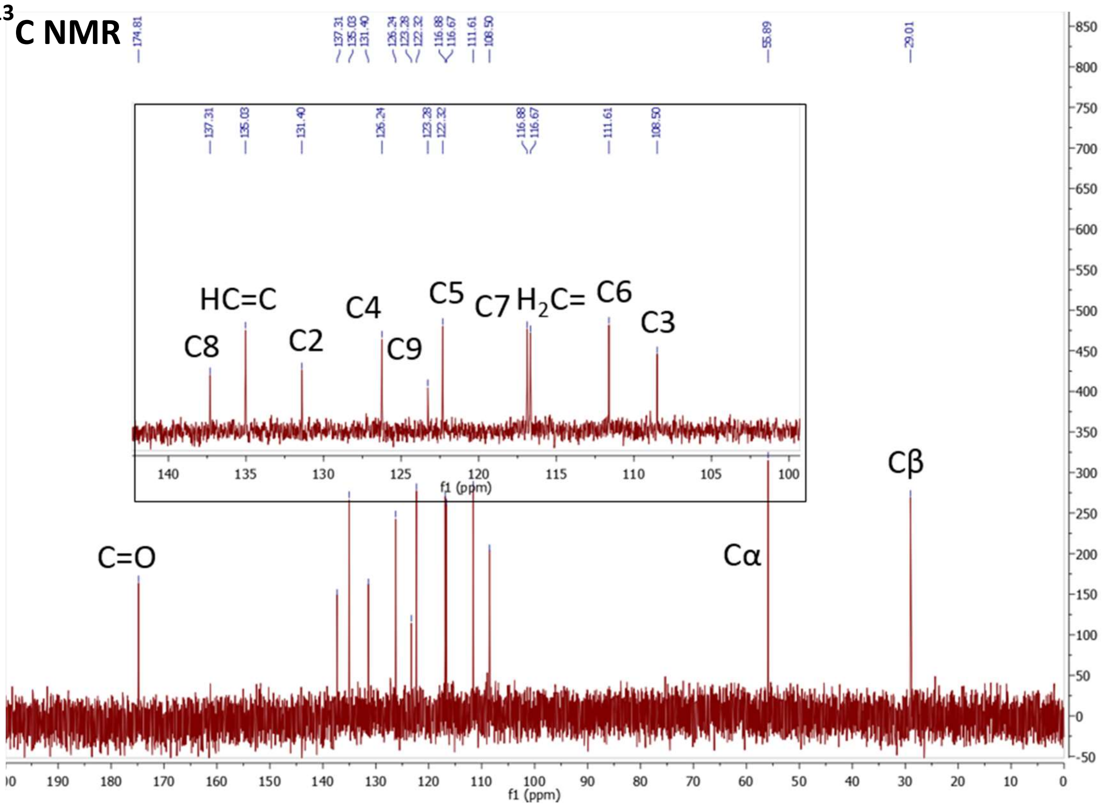

^{1}H NMR

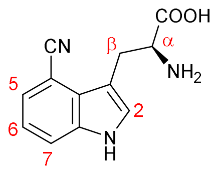

(**18**)

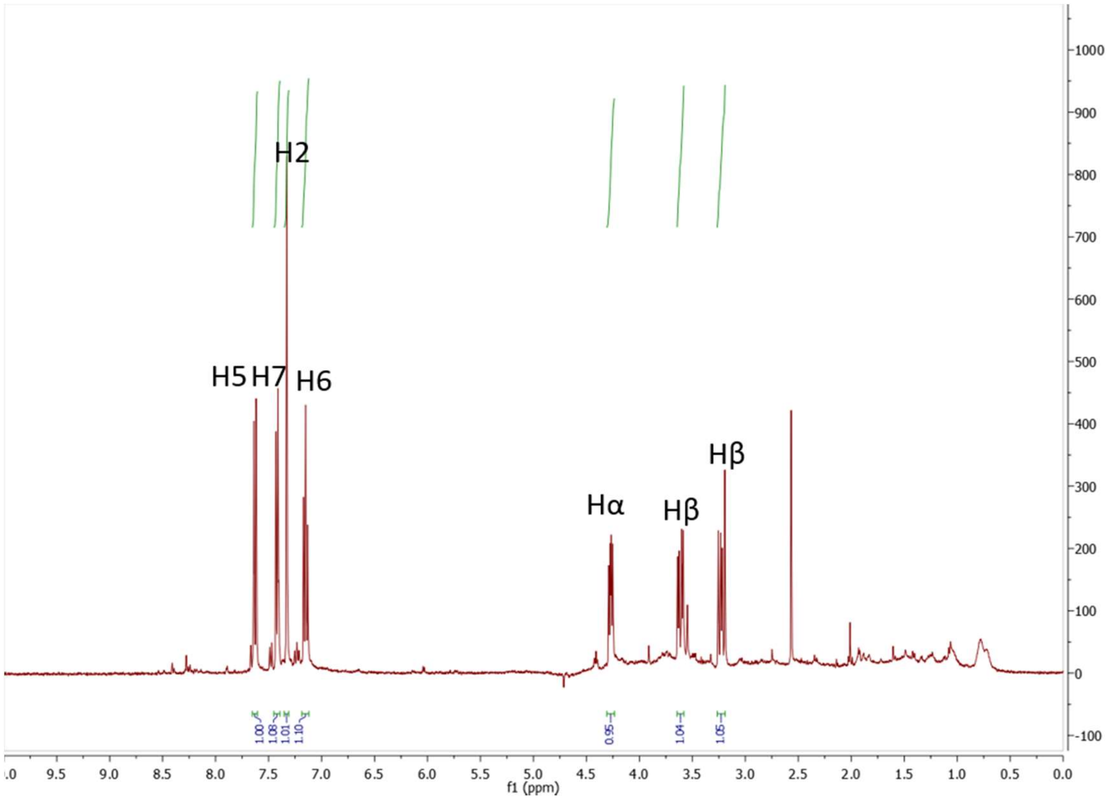

^{13}C NMR

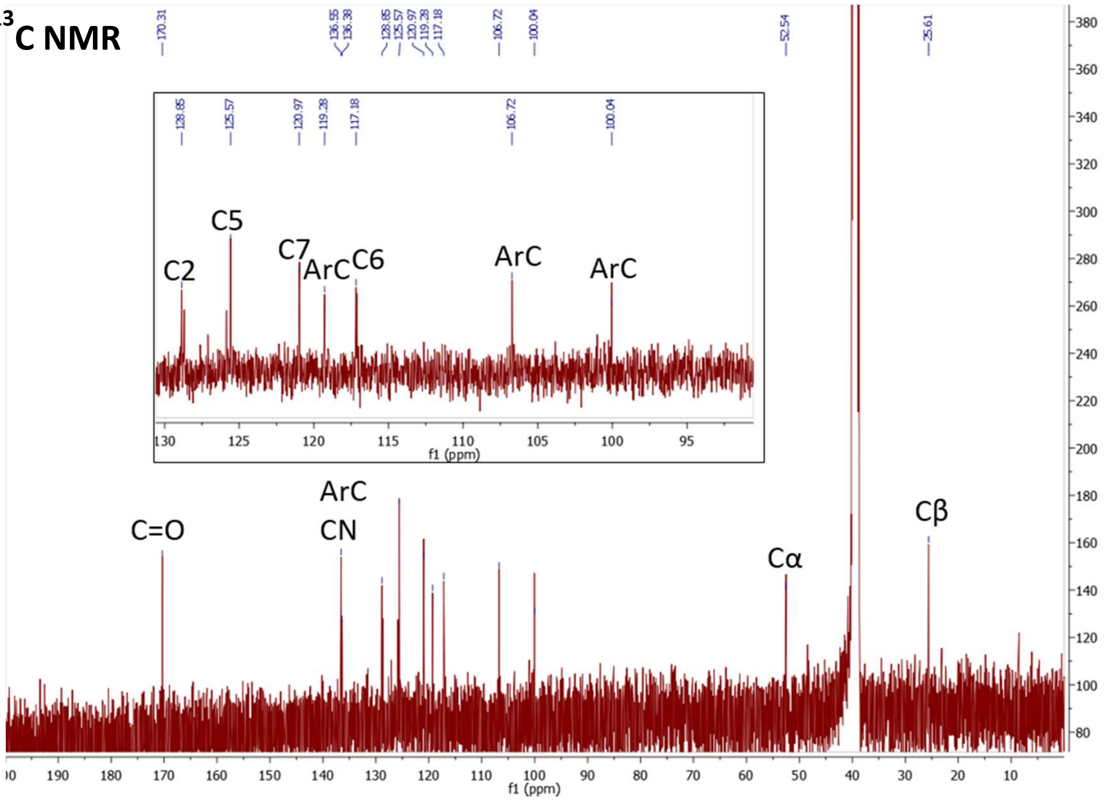

^{1}H NMR

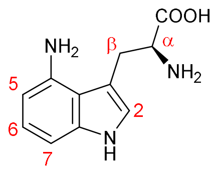

(**19**)

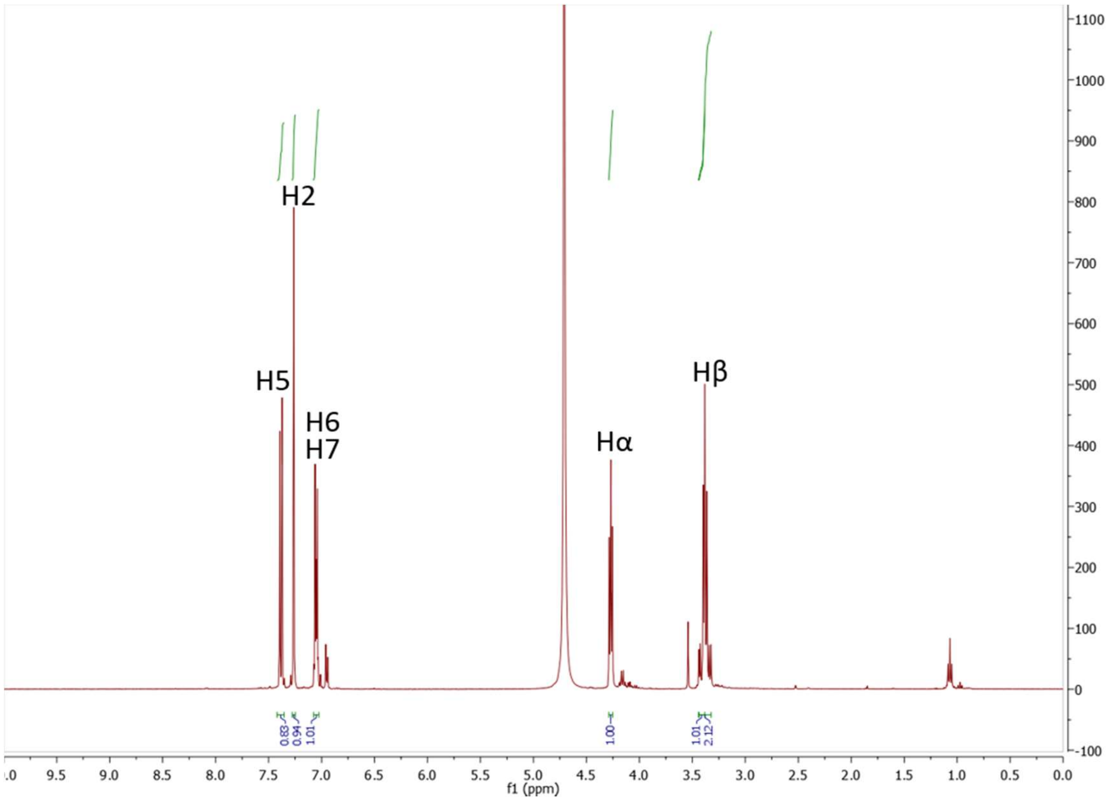

^{13}C NMR

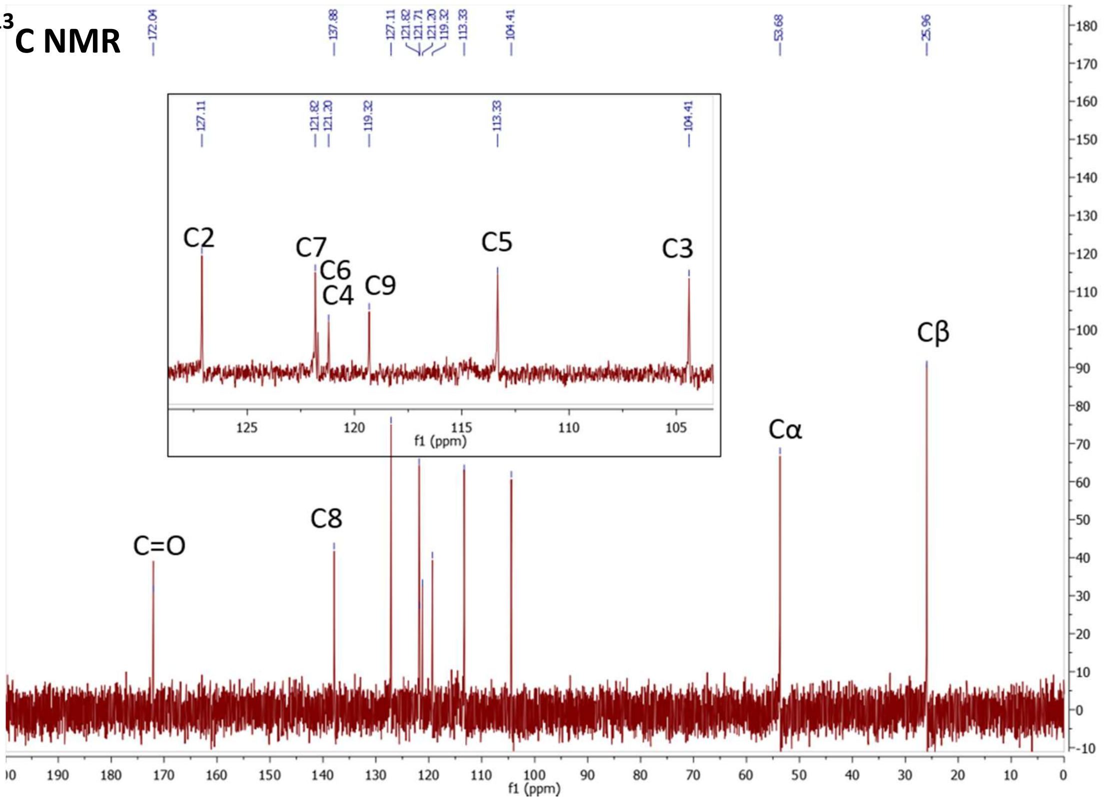

^{1}H NMR

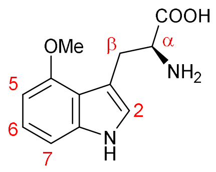

(**20**)

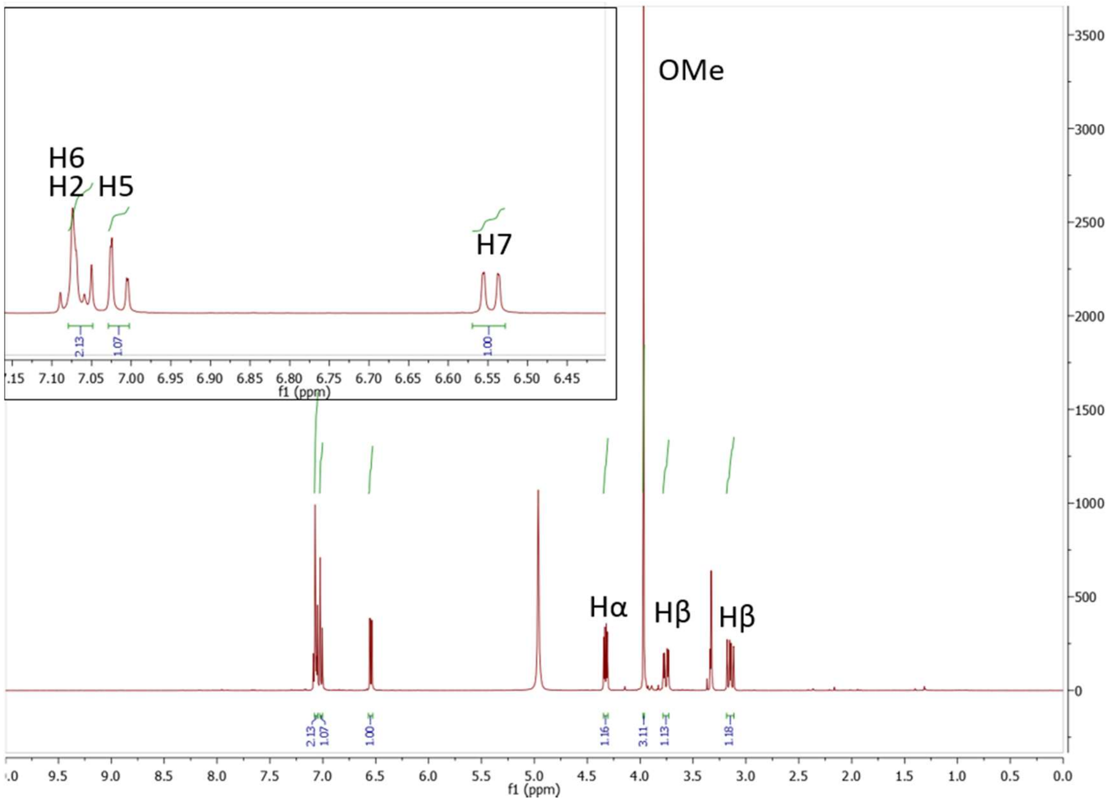

^{13}C NMR

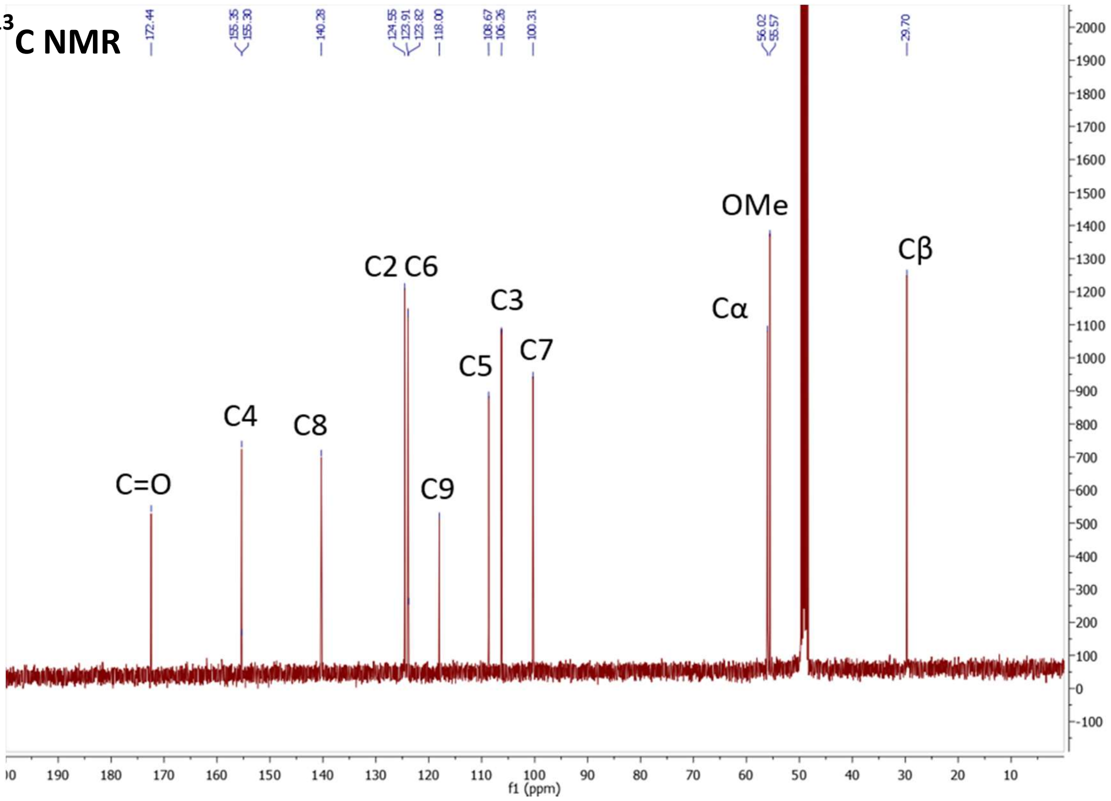

^{1}H NMR

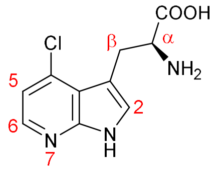

(**21**)

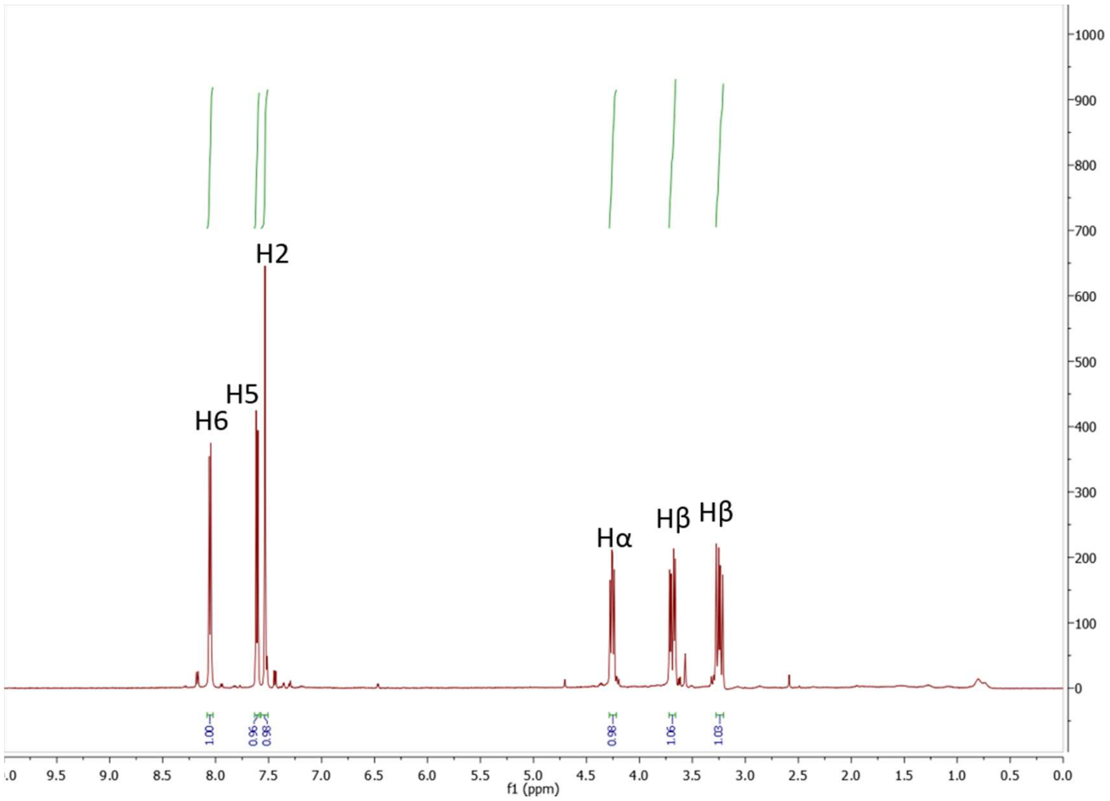

^{13}C NMR

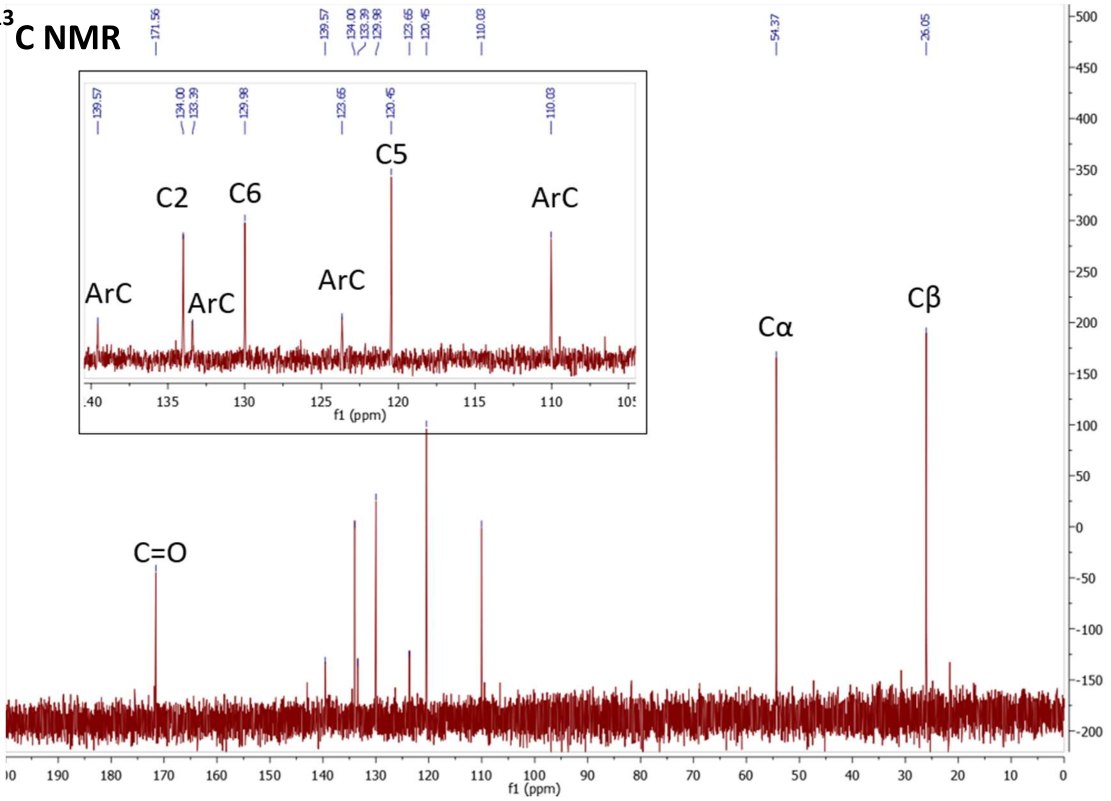

^{1}H NMR

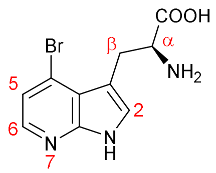

(**22**)

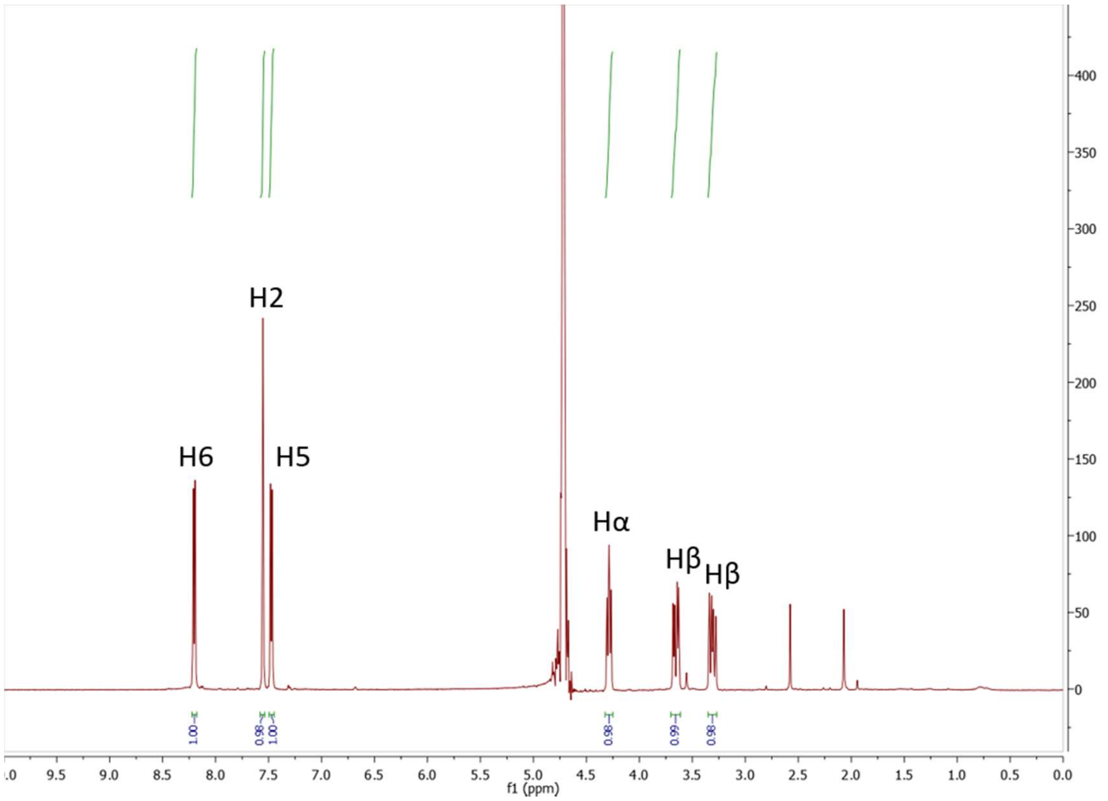

^{13}C NMR

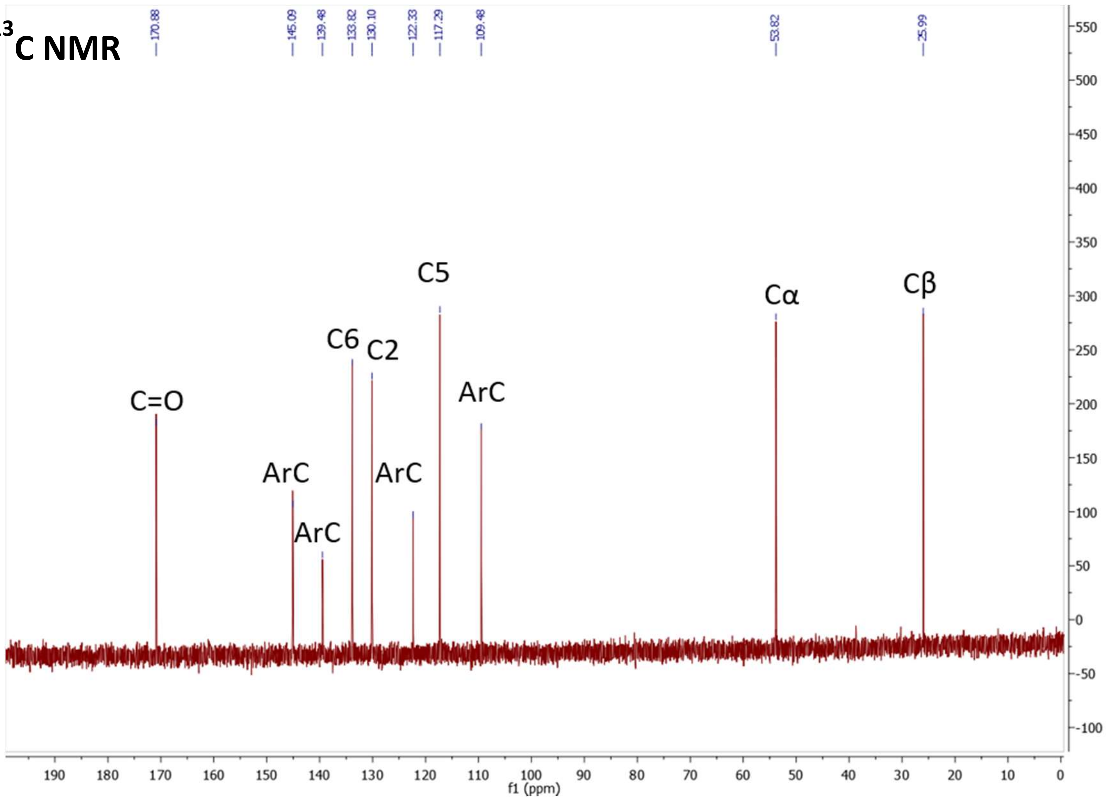

^{1}H NMR

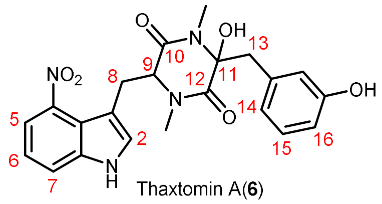

Thaxtomin A(**6**)

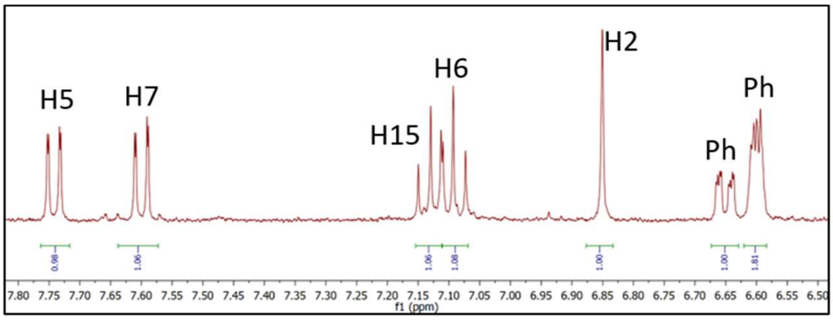

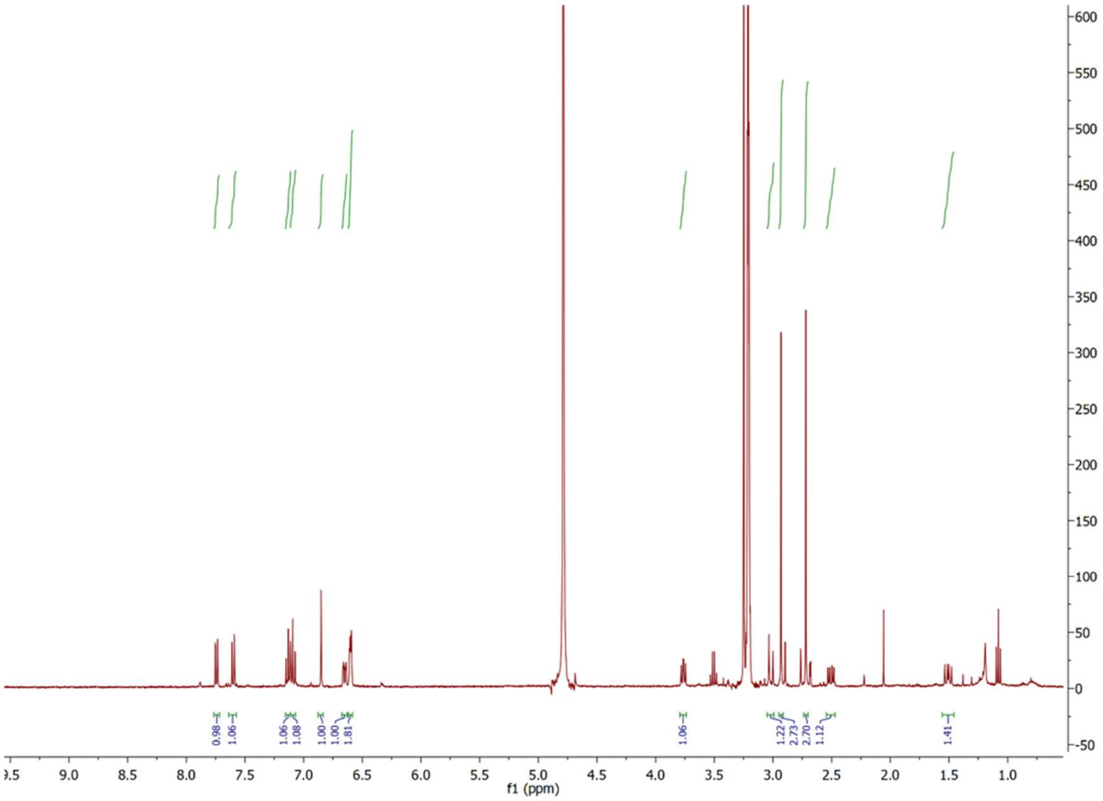

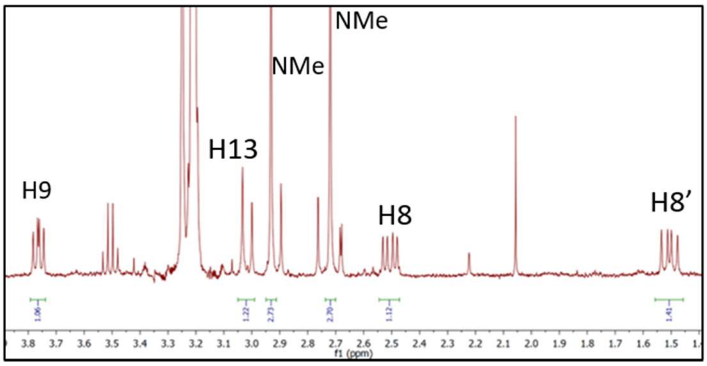

^{1}H NMR

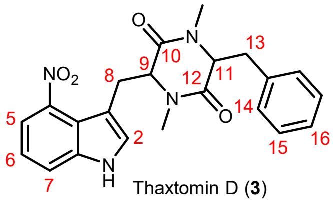

Thaxtomin D (**3**)

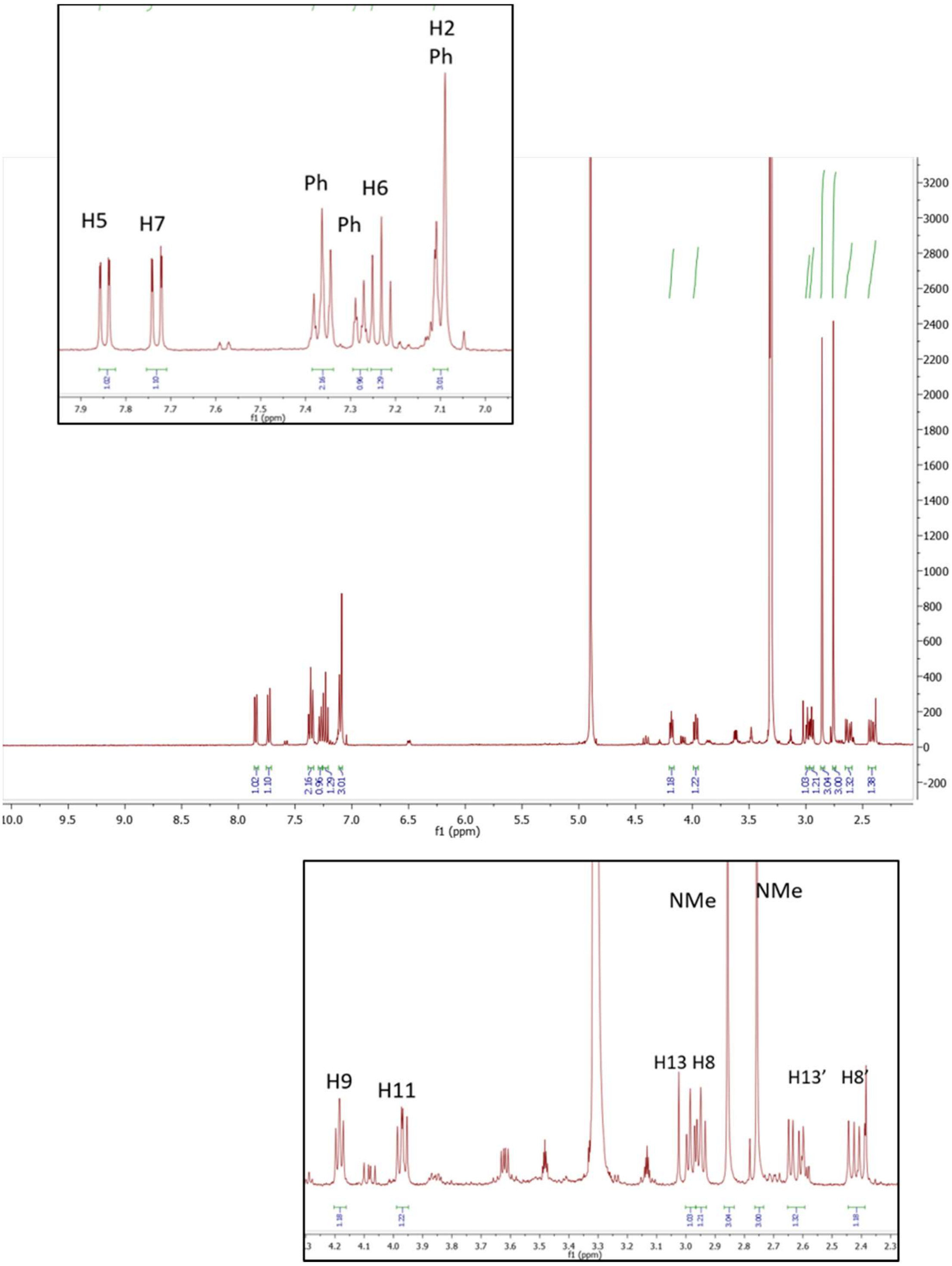

COSY expansion

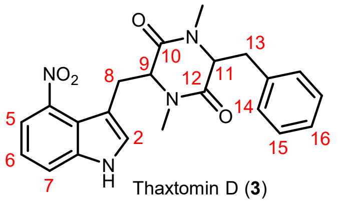

Thaxtomin D (**3**)

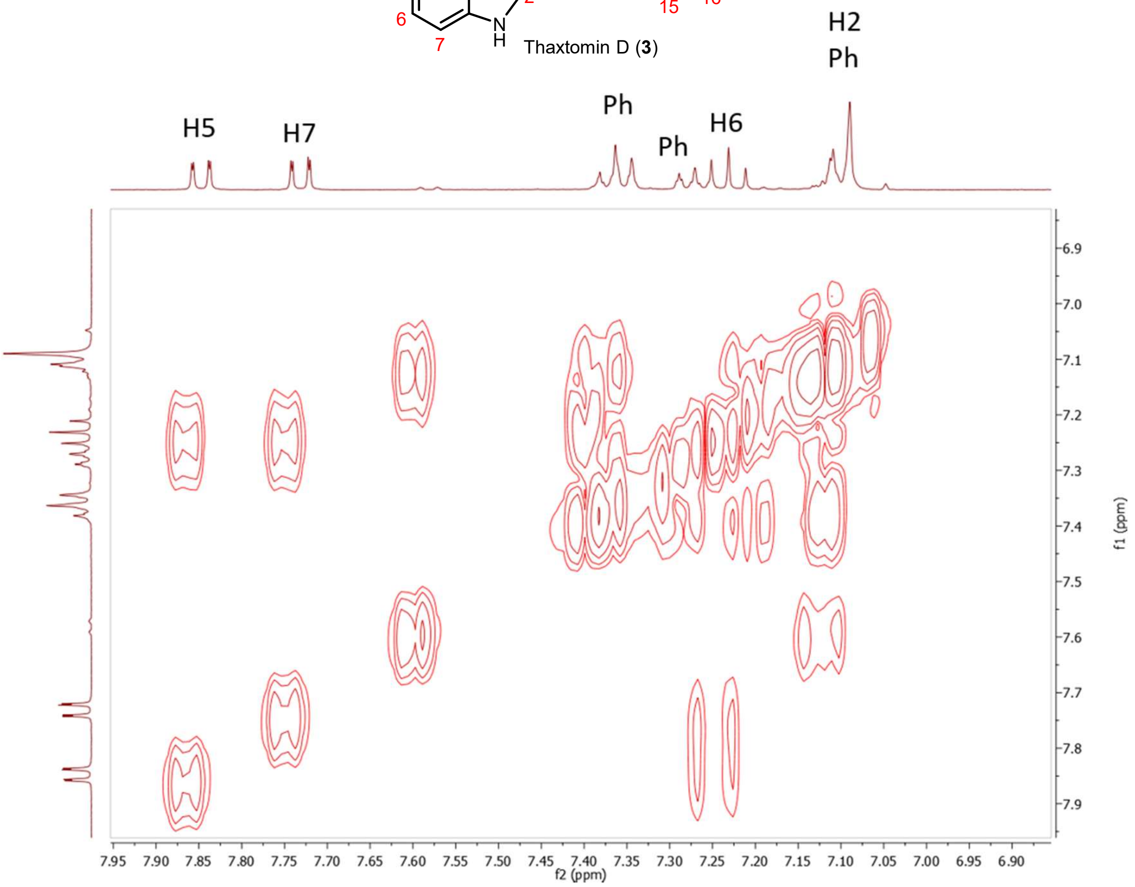

COSY expansion

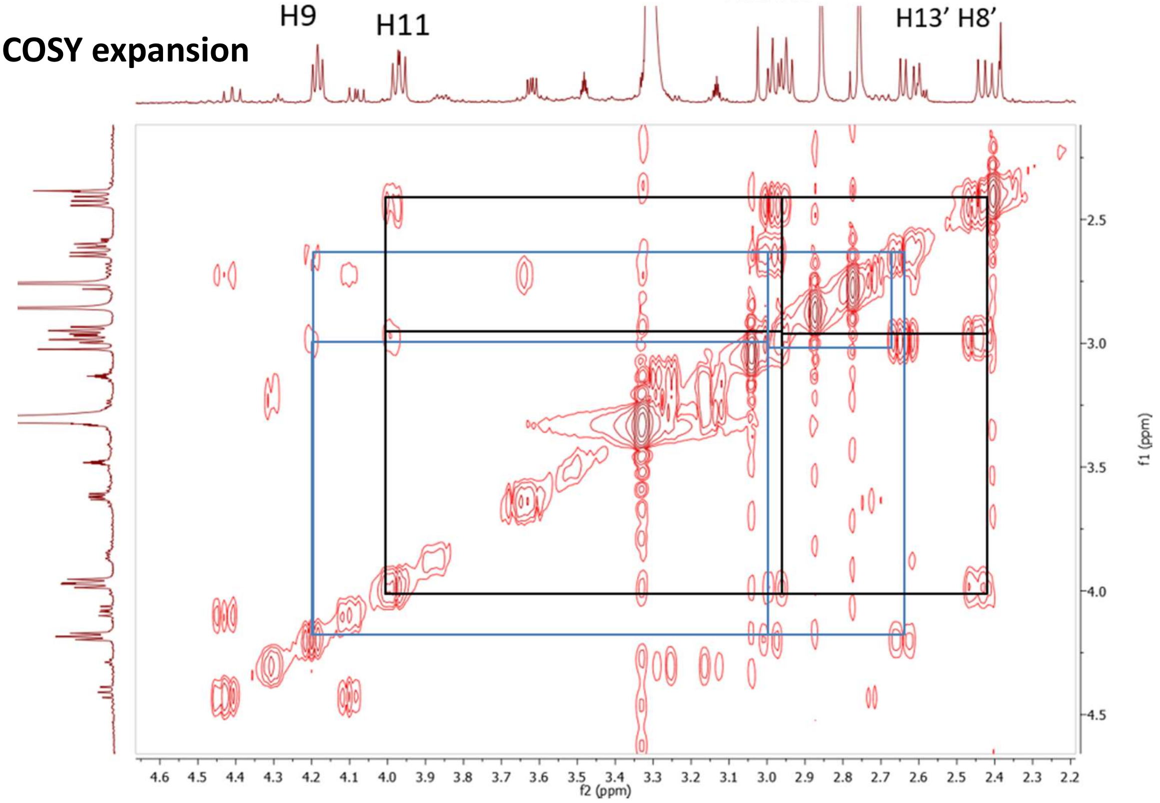

^{1}H NMR

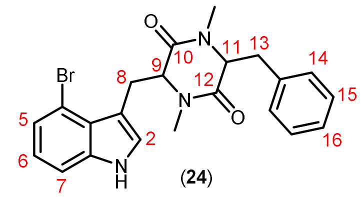

(**24**)

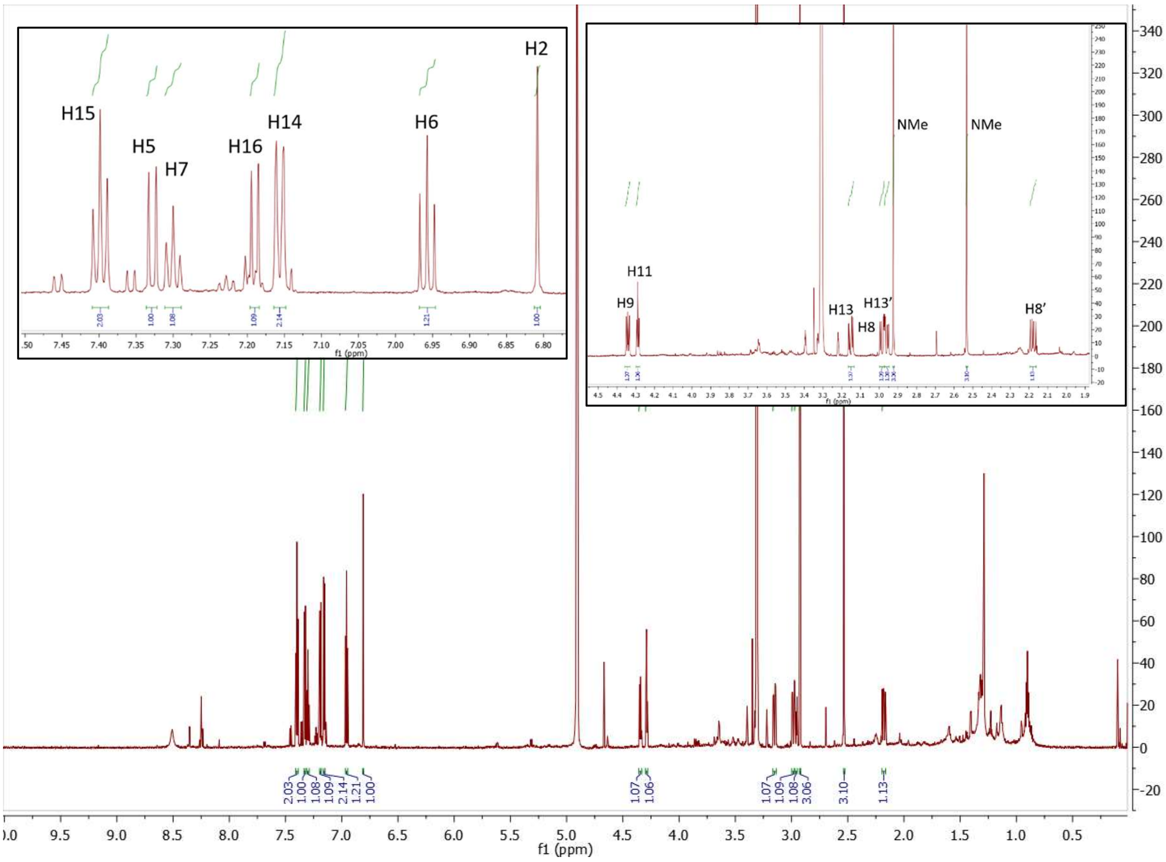

COSY

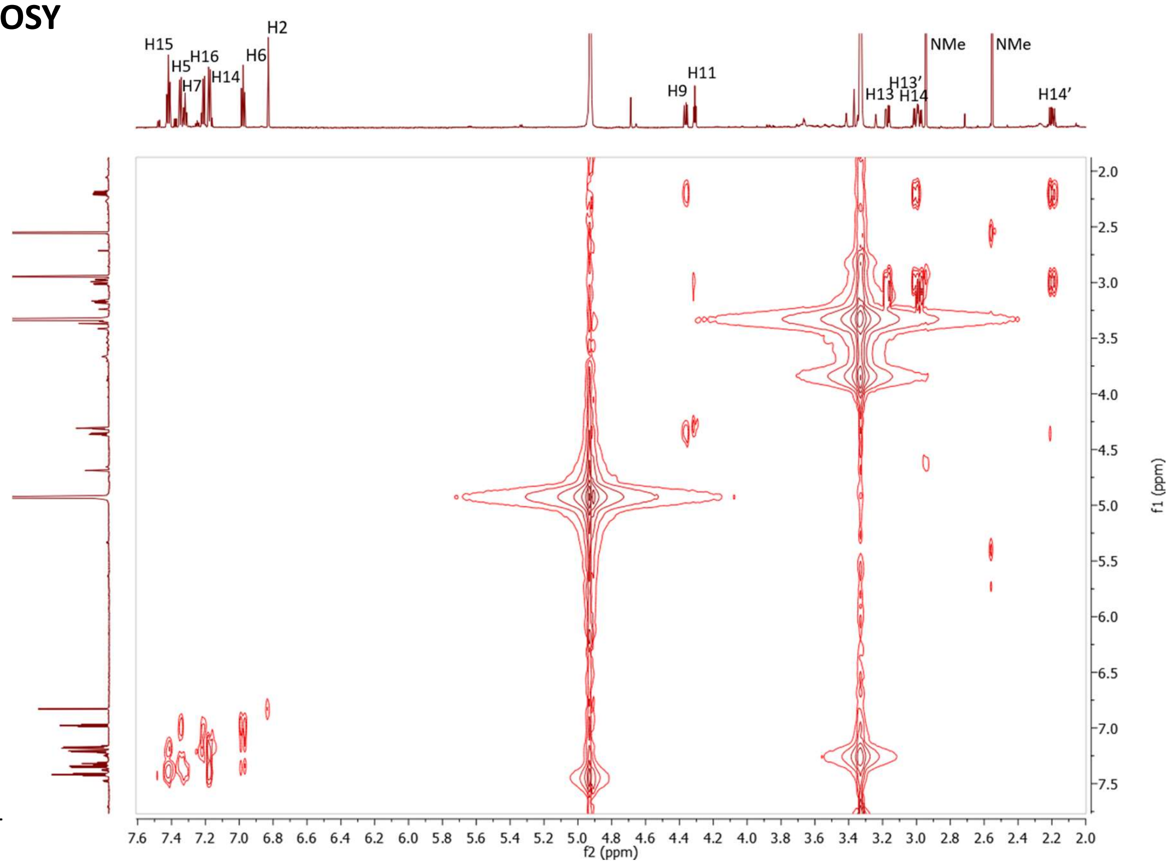

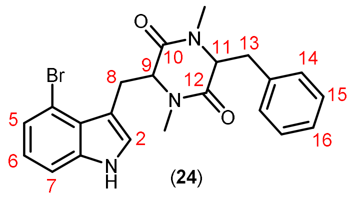

(**24**)

COSY expansion

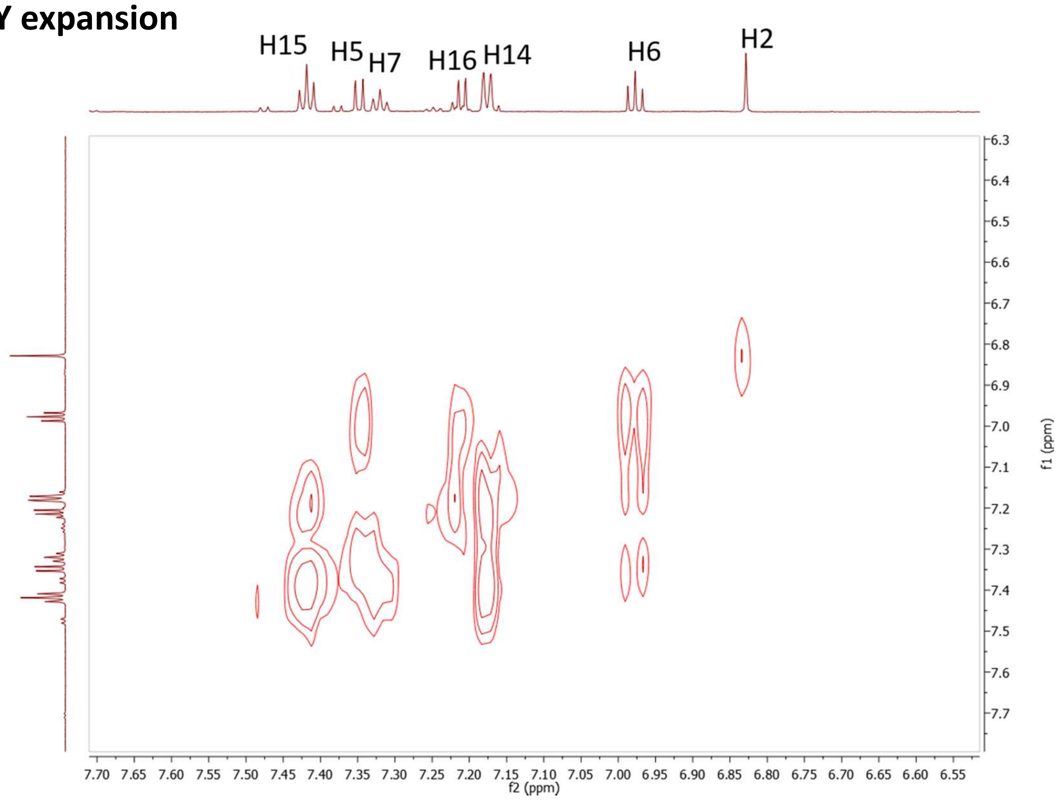

COSY expansion

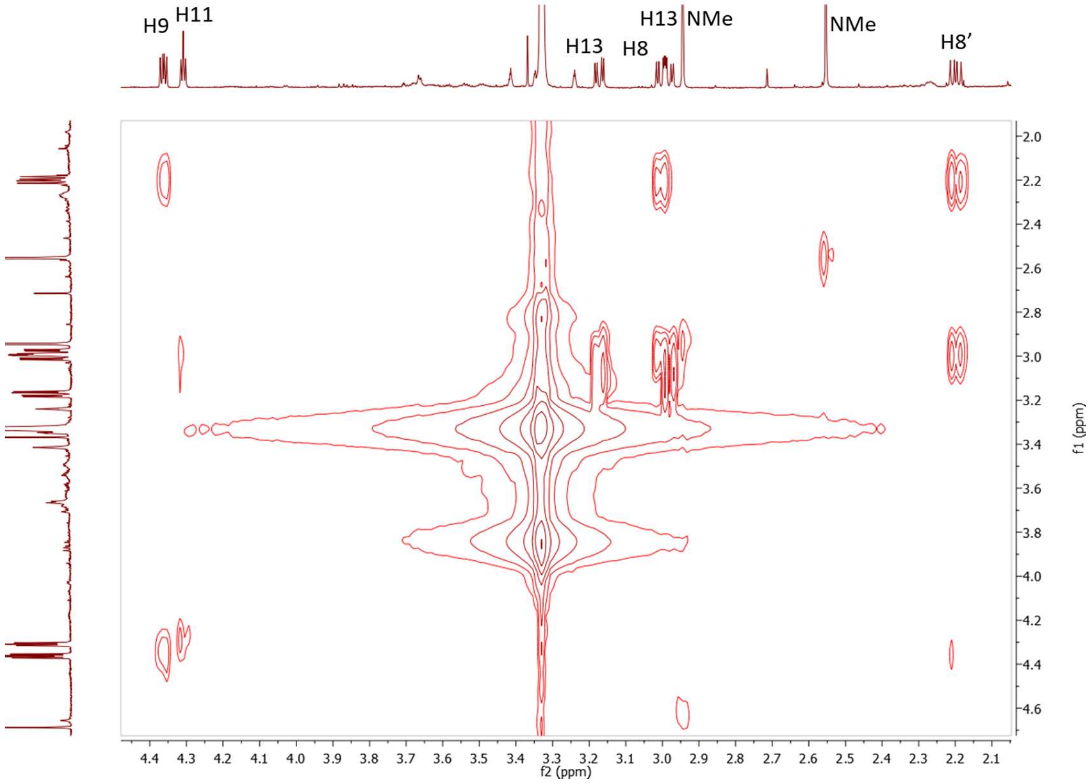

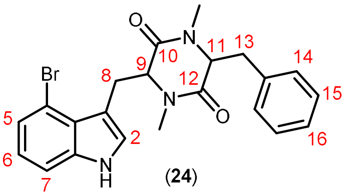

(24)

^{13}C NMR

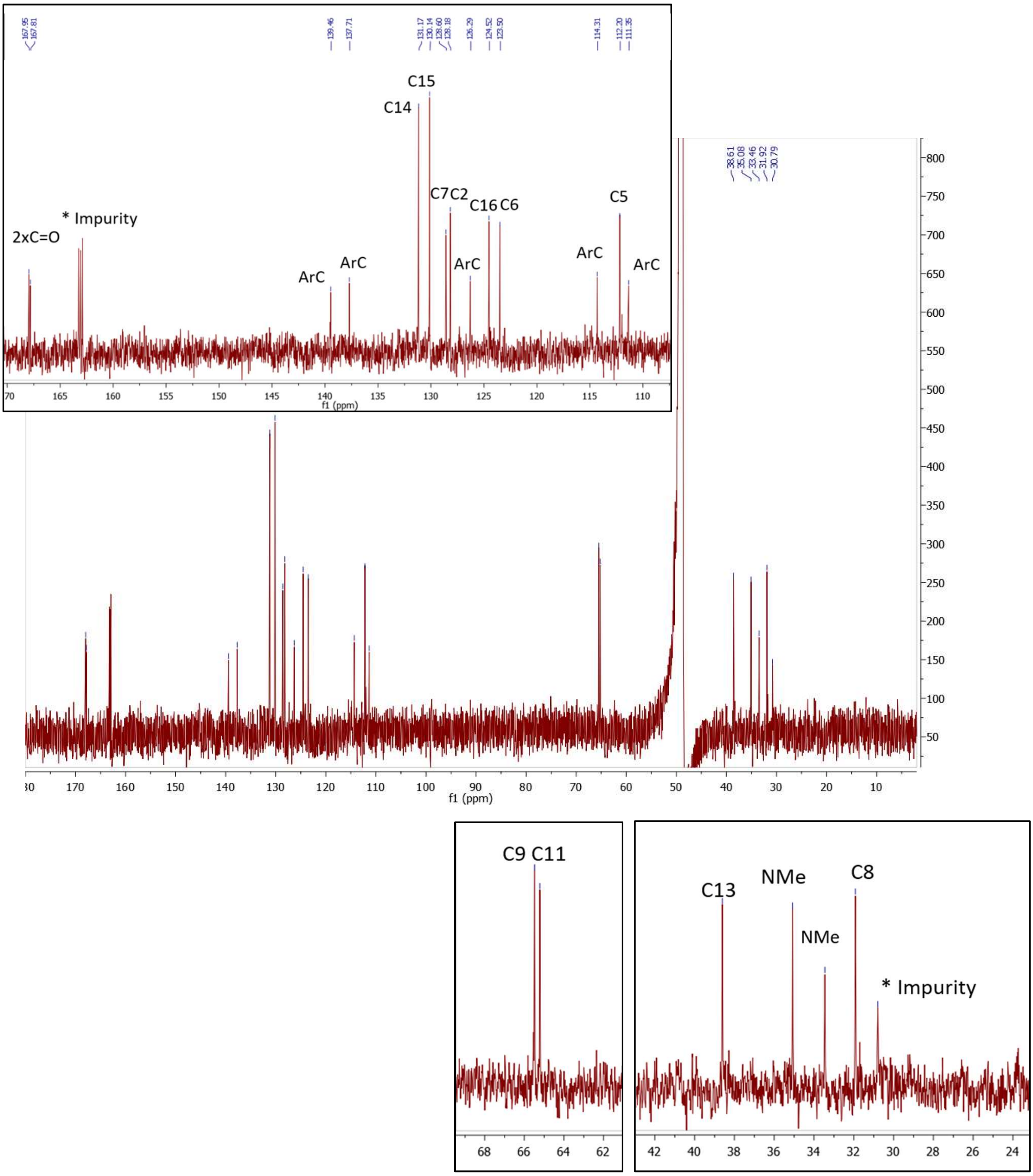

167.95
167.81
139.46
137.71
131.17
130.14
128.60
128.18
126.29
124.52
123.50
114.31
112.20
111.35
2xC=O
* Impurity
ArC
ArC
C14
C15
C7 C2
C16 C6
ArC
ArC
C5
ArC
38.61
35.08
33.46
31.92
30.79
f1 (ppm)
C9 C11
C13
NMe
NMe
C8
* Impurity

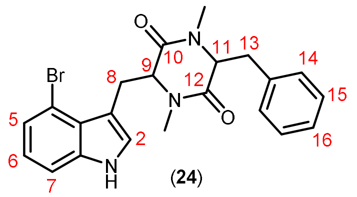

(**24**)

HSQC

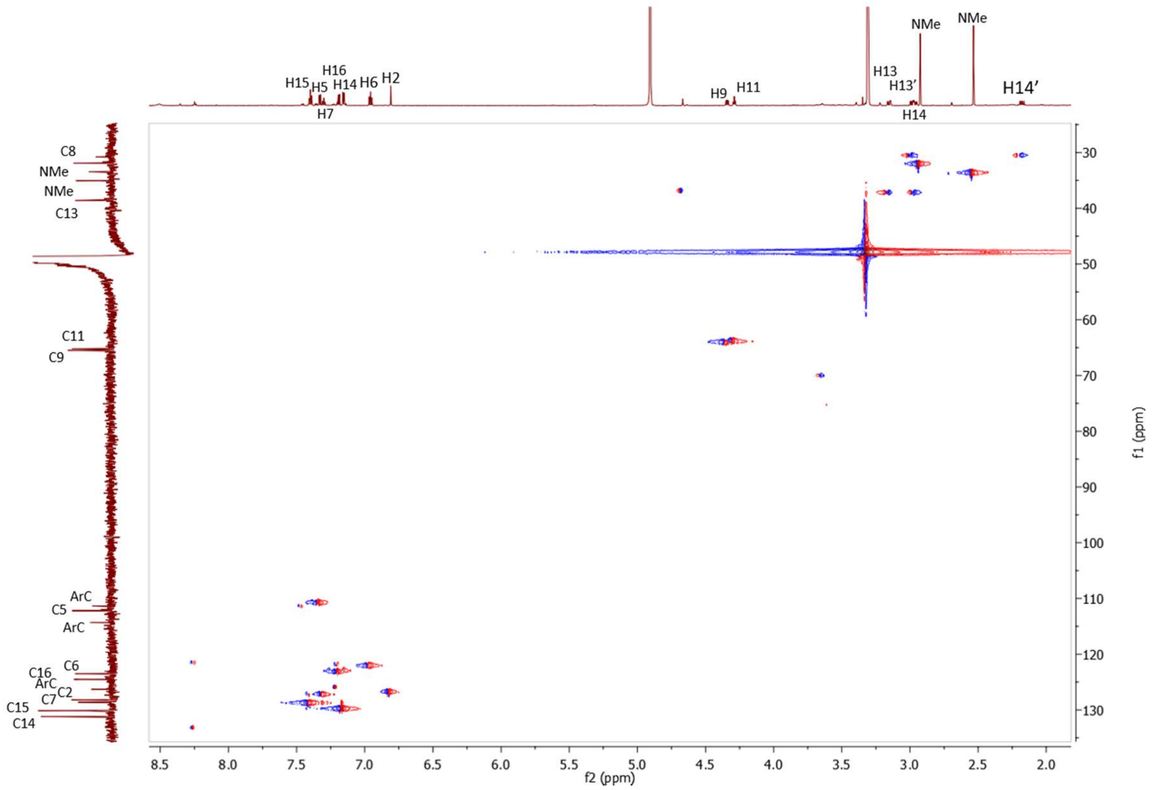

HSQC expansion

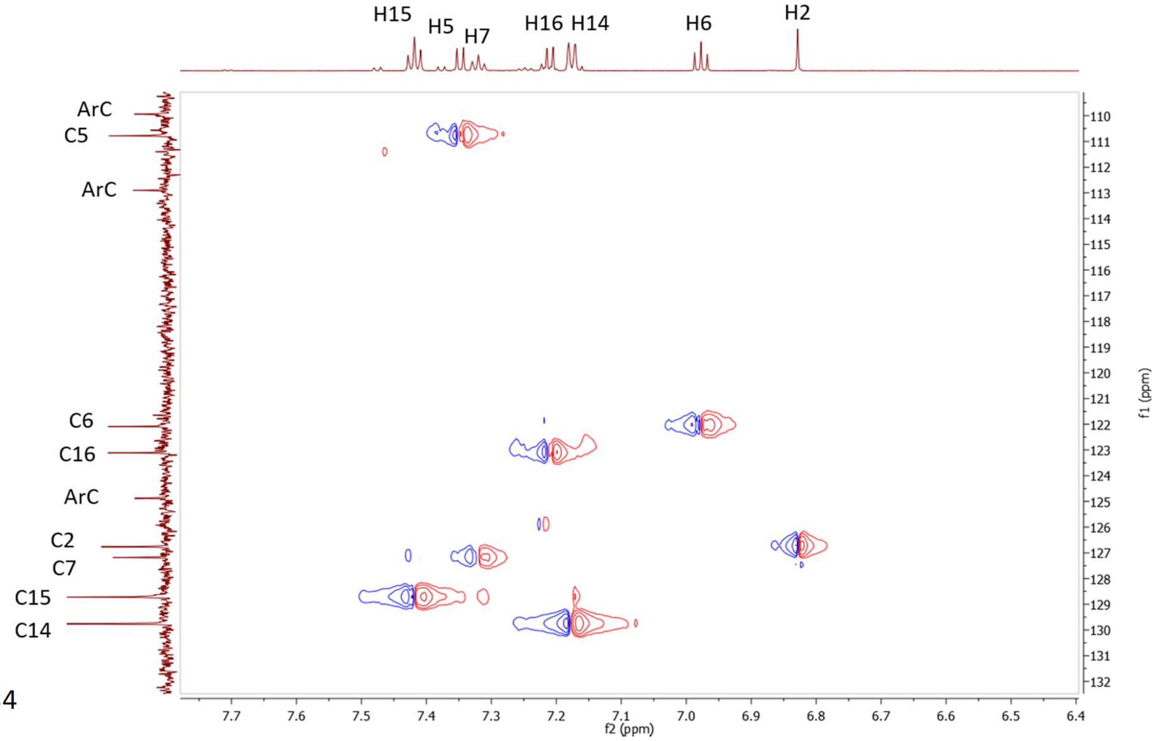

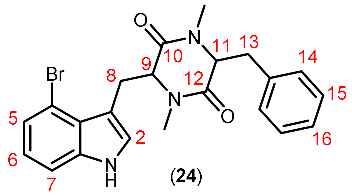

(24)

HSQC expansion

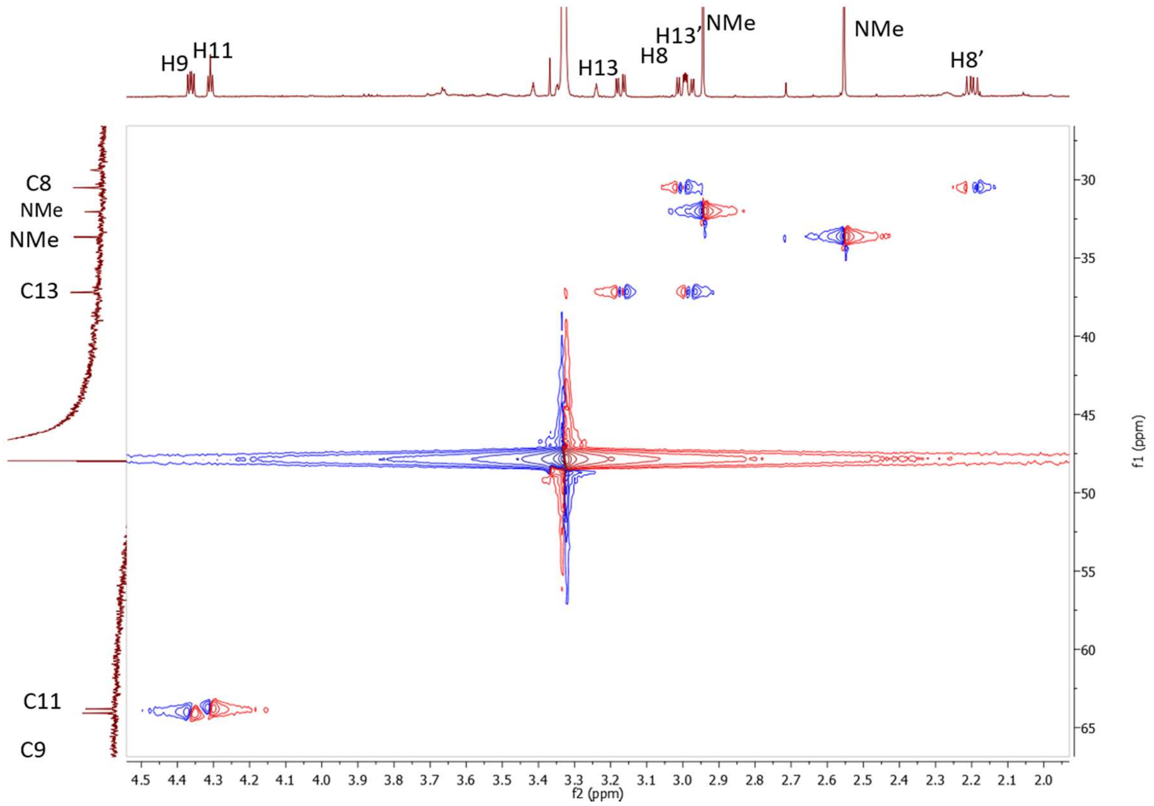

HMBC

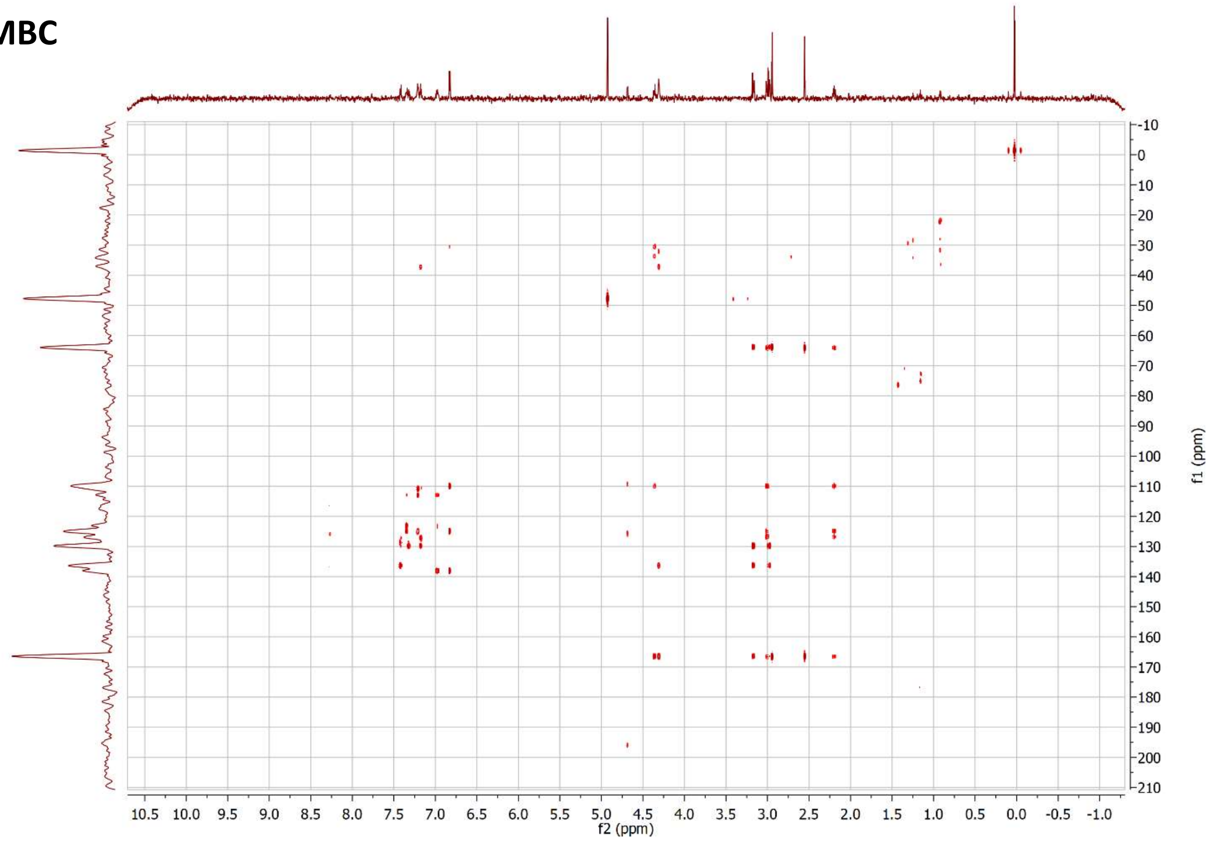

Supplementary References

[1] E. G. Johnson, M. V. Joshi, D. M. Gibson, R. Loria. *Physiol Mol. Plant. Path.* **2007,** *71*, 18-25

Abbreviated References from Manuscript

[5] A. Wlodek, S. G. Kendrew, N. J. Coates, A. Hold, J. Pogwizd, S. Rudder, L. S. Sheehan, S. J. Higginbotham, A. E. Stanley-Smith, T. Warneck, M. Nur-E-Alam, M. Radzom, C. J. Martin, L. Overvoorde, M. Samborskyy, S. Alt, D. Heine, G. T. Carter, E. I. Graziani, F. E. Koehn, L. McDonald, A. Alanine, R. M. Rodriguez Sarmiento, S. K. Chao, H. Ratni, L. Steward, I. H. Norville, M. Sarkar-Tyson, S. J. Moss, P. F. Leadley, B. Wilkinson, M. A. Gregory. *Nat. Commun.*, **2017**, *8*.

[7] J. Fu, X. Bian, S. Hu, H. Wang, F. Huang, P. M. Seibert, A. Plaza, L. Xia, R. Müller, A. F. Stewart, Y. Zhang. *Nat. Biotechnol.*, **2012,** *30,* 440-446.

[11] B. Zetsche, M. Heidenreich, P. Mohanraju, I. Fedorova, J. Kneppers, E. M. DeGennaro, N. Winblad, S. R. Choudhury, O. O. Abudayyeh, J. S. Gootenberg, W. Y. Wu, D. A. Scott, K. Severinov, J. van der Oost, F. Zhang, F. *Nat. Biotechnol.*, **2017,** *35,* 31-34.

[24] S. M. Barry, J. A. Kers, E. G. Johnson, L. Song, P. R. Aston, B. Patel, S. B. Krasnoff, B. R. Crane, D. M. Gibson, R. Loria, G. L. Challis. *Nat. Chem. Biol.*, **2012,** *8,* 814-816.

[35] H. Zhang, X. Ning, H. Hang, X. Ru, H. Li, Y. Li, L. Wang, X. Zhang, S. Yu, Y. Qiao, X. Wang, P. G. Wang. *Org. Lett.*, **2013,** *15,* 5670-5673.

[36] H. Zhang, Q. Wang, X. Ning, H. Hang, J. Ma, X. Yang, X. Lu, J. Zhang, Y. Li, C. Niu, H. Song, X. Wang, P. G. Wang. *J. Agric. Food Chem*, **2015,** *63,* 3734-3741.
